# Supplementary material for: Vitamin D deficiency or supplementation and the risk of human herpesvirus infections or reactivation: a systematic review protocol
Source: BMJ Open. 2019 Oct 7;9(10):e031867. doi: 10.1136/bmjopen-2019-031867 (PMC6797410; doi:10.1136/bmjopen-2019-031867)
Supplement: Supplementary data [file bmjopen-2019-031867supp001.pdf]

Supplementary Table 1: List of oral vitamin D analogues

Name	Indication	Brand name
Paricalcitol (19-nor-1α,25(OH)2D2)	Secondary hyperparathyroidism	Zemplar® (Abbott)
Doxercalciferol (1α(OH)D2)	Secondary hyperparathyroidism	Hectorol® (Genzyme corp)
Falecalcitriol (26,27 F6-1α,25(OH)2D3)	Secondary hyperparathyroidism (Japan only)	Fulstan® (Dainippon Sumitomo) and Hornel® (Taisho Yakuhin)
Maxacalcitol (22oxa-1α,25(OH)2D3)	Secondary hyperparathyroidism and psoriasis (Japan only)	Oxarol® (Chugai Pharmaceutical)
Eldecalcitol (2β-(3-hydroxypropoxy)-1α,25(OH)2D3)	Osteoporosis (Japan only)	Edirol® (Chugai Pharmaceutical)

Supplementary Table 2: Search strategies in Medline (Ovid)

Item	Search Terms
Herpesviruses	
1	(HHV adj1 ("1" or "2" or "3" or "4" or "5" or "6" or "7" or "8")) or (HHV?1 or HHV?2 or HHV?3 or HHV?4 or HHV?5 or HHV?6 or HHV?7 or HHV?8)
2	exp Herpesviridae Infections/ or exp Herpesviridae/
3	herpes* or herpesvir*
4	exp Herpes Simplex/
5	herpes simplex
6	HSV-1 or HSV?1
7	Cold sore*
8	exp Herpes Labialis/
9	Herpes Labialis
10	Herpes* adj2 Labial*
11	Hsv-2 or HSV?2 or (genit* herpes* or genit* sores)
12	exp Herpes Zoster/
13	herpes zoster
14	vz virus* or VZV
15	varicella?
16	chickenpox or Chicken?pox
17	shingle*
18	neuralgia adj1 post?herpetic)
19	Epstein-Barr virus* or (burkitt lymphoma adj2 virus*) or e?b virus* or ((eb or e-b) adj virus*) or ebv or Epstein?barr virus* or infectious mononucleosis or glandular fever
20	Cytomegalovirus* or CMV
21	salivary gland virus* or hblv or betaherpesvirus or b-lymphotropic virus* or cihhv or icihhv or Roseolovirus or Roseola Infantum or Exanthema Subitum
22	Kaposi* sarcoma-associated herpesvir* or KSHV or Primary Effusion Lymphoma
23	ac?clovir or Zovirax or valac?clovir or valtrex or famc?clovir or famvir or penc?clovir or ganc?clovir or cidofovir or foscarnet* or valganc?clovir or lubocavir or brivudin or Docosanol or Sorivudine or Idoxuridine or Trifluridine
24	1 or 2 or 3 or 4 or 5 or 6 or 7 or 8 or 9 or 10 or 11 or 12 or 13 or 14 or 15 or 16 or 17 or 18 or 19 or 20 or 21 or 22 or 23
Vitamin D	
25	exp Vitamin D/ or exp Vitamin D Deficiency/
26	(vitamin* or hydroxyvitamin* or dihydroxyvitamin*) adj1 d*
27	c?olecalciferol* or calciol* or calcitriol* or ergocalciferol* or calcifediol* or calcid?ol* or calciferol* or ercalcidiol* or ercalcitriol* or hydroxyergocalciferol* or hydroxycalciferol* or hydroxyc?olecalciferol* or dihydroxyc?olecalciferol* or ergosterol
28	(dihydrotachyst* or alphacalcid?ol or alfacalcid?ol or Falecalcitriol* or Maxacalcitol* or Oxacalcitriol* or Paricalcitol* or Lexacalcitol* or Seocalcitol* or Calcipotriol* or vitamin D analogue*
29	exp Familial Hypophosphatemic Rickets/ or exp Rickets, Hypophosphatemic/ or exp Rickets/
30	Rickets
31	rachitides
32	rachitis
33	25 or 26 or 27 or 28 or 29 or 30 or 31 or 32
34	24 and 33

Supplementary Table 3: modified keywords for grey literature search

Search Concept	Keywords Groups
Herpesviruses	Group 1: herpesvir* OR herpes* OR HHV* OR HSV* OR Cold sore* OR varicella* OR shingle*<br>Group 2: chickenpox OR post-herpetic neuralgia OR Burkitt lymphoma OR EBV OR Epstein-Barr<br>Group 3: Cytomegalovirus* or CMV OR Kaposi* sarcoma-associated herpesvir* or KSHV OR famc*
Vitamin D	Group 4: "vitamin D" or hydroxyvitamin* or dihydroxyvitamin*<br>Group 5: calcitriol* OR cholecalciferol OR ergocalciferol* OR Calcidol<br>Group 6: calcifer* OR ergosterol OR rickets OR Calcipotriol* OR vitamin D analogue
Combination of concepts	(Group 1, Group 2, Group 3) x (Group 4, Group 5, Group 6)

Supplementary Table 4: Domains of risk of bias assessment

Domains	Explanation
Confounders	Not adjusted for confounders
Selection bias	Intervention studies: random sequence generation and allocation concealment<br>Observational studies: and participation bias, selection of participants, and selection of controls
Misclassification of exposure and outcomes	Differential: recall bias, observer bias<br>Non-differential: ascertainment bias
Performance bias and detection bias	Blinding of participants, personnel and outcome assessment
Attrition bias	Incomplete outcome data
Bias due to Missing Data	Differential loss to follow up and exclusion of individuals with missing data
Reporting bias	Selective reporting
Reverse causation	Defined outcomes prior to the exposures
