# Supplementary material for: Vitamin D deficiency or supplementation and the risk of human herpesvirus infections or reactivation: a systematic review protocol
Source: BMJ Open. 2019 Oct 7;9(10):e031867. doi: 10.1136/bmjopen-2019-031867 (PMC6797410; doi:10.1136/bmjopen-2019-031867)
Supplement: Author's manuscript [file bmjopen-2019-031867.draft_revisions.pdf]

BMJ Open

BMJ Open is committed to open peer review. As part of this commitment we make the peer review history of every article we publish publicly available.

When an article is published we post the peer reviewers' comments and the authors' responses online. We also post the versions of the paper that were used during peer review. These are the versions that the peer review comments apply to.

The versions of the paper that follow are the versions that were submitted during the peer review process. They are not the versions of record or the final published versions. They should not be cited or distributed as the published version of this manuscript.

BMJ Open is an open access journal and the full, final, typeset and author-corrected version of record of the manuscript is available on our site with no access controls, subscription charges or pay-per-view fees (http://bmjopen.bmj.com).

If you have any questions on BMJ Open's open peer review process please email info.bmjopen@bmj.com

BMJ Open

Vitamin D and human herpesviruses infections: a systematic review protocol

Journal:	*BMJ Open*
Manuscript ID	bmjopen-2019-031867
Article Type:	Protocol
Date Submitted by the Author:	22-May-2019
Complete List of Authors:	Lin, Liang-Yu; London School of Hygiene and Tropical Medicine Faculty of Epidemiology and Population Health, Department of Non-communicable Disease Epidemiology<br>Bhate, Ketaki; London School of Hygiene and Tropical Medicine Faculty of Epidemiology and Population Health, Department of Non-communicable Disease Epidemiology<br>Forbes, Harriet; London School of Hygiene and Tropical Medicine Faculty of Epidemiology and Population Health, Department of Non-communicable Disease Epidemiology<br>Smeeth, Liam; London School of Hygiene and Tropical Medicine Faculty of Epidemiology and Population Health, Department of Non-communicable Disease Epidemiology<br>Warren-Gash, Charlotte; London School of Hygiene and Tropical Medicine Faculty of Epidemiology and Population Health, Department of Non-communicable Disease Epidemiology<br>Langan, Sinead; London School of Hygiene and Tropical Medicine Faculty of Epidemiology and Population Health, Department of Non-communicable Disease Epidemiology
Keywords:	systematic review, herpesviridae, vitamin D deficiency, vitamin D supplementation

SCHOLARONE™
Manuscripts

Title: Vitamin D and human herpesviruses infections: a systematic review protocol

Authors

Contact

Dr Liang-Yu Lin[1]. LSHTM liang-yu.lin@lshtm.ac.uk ORCID: https://orcid.org/0000-0003-4720-6738

Dr Ketaki Bhate[1]. LSHTM Ketaki.Bhate@lshtm.ac.uk

ORCID: https://orcid.org/0000-0001-5509-4428

Dr Harriet Forbes[1]. LSHTM Harriet.Forbes@lshtm.ac.uk

ORCID: https://orcid.org/0000-0001-6888-2212

Professor Liam Smeeth[1]. LSHTM Liam.Smeeth@lshtm.ac.uk

ORCID: https://orcid.org/0000-0002-9168-6022

Professor Sinéad M Langan[1]*. LSHTM Sinead.Langan@LSHTM.ac.uk

ORCID: https://orcid.org/0000-0002-7022-7441

Dr Charlotte Warren-Gash[1]*. LSHTM Charlotte.Warren-Gash1@lshtm.ac.uk

ORCID: https://orcid.org/0000-0003-4524-3180

* The senior authors contributed equally to this work

Authors' Affiliations:

1. Faculty of Epidemiology & Population Health, London School of Hygiene and Tropical Medicine, London, UK

For peer review only

Correspondence to: Professor Sinéad M Langan

LSHTM, Keppel Street, London, WC1E 7HT, United Kingdom

Tel: 0207 9272680, Sinead.Langan@LSHTM.ac.uk

Word count: 1948

Keywords: systematic review, herpesviridae, vitamin D deficiency, vitamin D supplementation

For peer review only

ABSTRACT:

Introduction:

Herpesviruses induce lifelong latent infections in humans and may reactivate as the immune system deteriorates. Recent studies have suggested that vitamin D, an essential element of bone health, may have some effect on immunomodulation. Studies have indicated that vitamin D may protect against infection, but investigations of its potential to prevent herpesvirus infection or reactivation are limited. We will review the current literature examining vitamin D deficiency and the risk of herpesvirus infections, and the protective effects of vitamin D against herpesvirus infections.

Methods and analysis:

A systematic review approach will address two research questions: 1. Do deficient/insufficient serum vitamin D levels increase the risk of herpesvirus infections, and 2. Does vitamin D supplementation protect against herpesvirus infections? We will include only intervention studies, cohort studies and case-control studies. We will use subject headings and keywords to search for synonyms of “vitamin D” AND “herpesviruses” in databases, including Medline, Embase, Global Health, Web of Science, Scopus, and Cochrane Central Register of Controlled Trials, and the grey literature databases Prevention Information & Evidence eLibrary, Open Grey, EThOS, and BASE. The references of the included articles and relevant systematic reviews will also be examined. Two reviewers will independently screen the study titles and abstracts, and examine the full texts to decide the final eligibility. They will independently extract data from the studies and assess bias using the Cochrane

For peer review only

Collaboration approach. The third researcher will solve any discrepancy in inclusion, data extraction and bias assessment. The results will be narratively synthesized; if an adequate number of studies is included and the homogeneity between studies is acceptable, a meta-analysis will be carried out. We will assess the quality of evidence using the Grading of Recommendations, Assessment, Development and Evaluation (GRADE) framework, and display the results in a summary of findings table.

Ethics and dissemination

Ethical review is not required for a systematic review. We will publish the results in a peer-review journal. Any amendments to the protocol will be recorded in the supplementary section.

PROSPERO registration number:

CRD42019130153

For peer review only

STRENGTHS AND LIMITATION OF THIS STUDY:

- This systematic review will be undertaken following the predefined population, exposure, comparator and outcome framework
- All available databases, including six major databases and four grey literature databases, will be searched to obtain all eligible studies.
- The summarized results will improve our understanding of the possible association between vitamin D and herpesvirus infection/reactivation.
- The number of sufficient eligible studies may be inadequate, or the included studies may not have adequate quality of evidence to answer the research questions.

For peer review only

INTRODUCTION

Rationale

Herpesviruses are a group of double-stranded DNA viruses that infect humans and some animals. After infecting their hosts, these viruses cannot be eradicated; instead, they establish latency and persist for life. As the host's immunity declines, these viruses can reactivate to induce various symptoms. There are eight human herpesviruses (Table 1)[1]. Reactivation of these viruses may lead to serious complications, such as herpetic keratitis (Herpes simplex virus 1, HSV-1), nasopharyngeal cancer (Epstein-Barr virus, EBV), herpes zoster and post-herpetic neuralgia (Varicella zoster virus, VZV), which may lead to blindness[2], malignancy[3], and increased financial burdens[4]. Consequently, investigating immunomodulatory factors associated with infection or reactivation of this virus family is important.

Vitamin D is mainly endogenously synthesized by the skin after sun exposure and can be supplied through dietary intake and supplementation. It plays an important role in absorbing calcium and phosphate, which are essential for bone health[5]. Recently, some studies have indicated that vitamin D may have potential immunomodulatory effects. In response to pathogen exposure, immune cells, such as monocytes or macrophages, upregulate vitamin D receptors and enzymes which catalyse vitamin D to synthesize anti-pathogenic peptides [6]. One meta-analysis indicated that among patients receiving dialysis due to chronic kidney diseases, patients with higher or normal serum vitamin D levels had a 39% lower risk of infection than those who were deficient in vitamin D (relative risk ratio: 0.61, 95% confidence interval: 0.41-0.89) [7]. Furthermore, vitamin D

For peer review only

supplementation may help to protect against viral infections. Another meta-analysis using original patient data from 25 randomized control trials showed that vitamin D supplementation reduced the risk of acute respiratory infections (odds ratio: 0.88, 95% confidence interval: 0.81-0.96)[8]. However, limited evidence exists concerning whether vitamin D is associated with herpesvirus infections.

In this study, we will comprehensively review studies of the effect of serum vitamin D levels or the use of oral vitamin D supplementation on infection with, or reactivation of any of the eight human herpesviruses.

OBJECTIVE

This review aims to explore the association between vitamin D and herpesviruses. The proposed systematic review will address two primary research questions:

1. Is serum vitamin D deficiency/insufficiency associated with an increased risk of infection with or reactivation of human herpesviruses?
2. Does oral vitamin D supplementation protect against infection with or reactivation of human herpesviruses?

For peer review only

METHODS

This study protocol will be reported according to the Preferred Reporting Items for Systematic review and Meta-Analysis Protocols (PRISMA-P)[9].

Eligibility Criteria

Study Design and Characteristics

To identify the possible causal association between vitamin D and herpesvirus infections, we will review observational studies, including cohort and case-control studies, and intervention studies, including randomized or non-randomized controlled trials. Descriptive studies, ecological studies, cross-sectional studies, case reports and case series will not be included. If a systematic review relevant to our topic is found, we will review its references.

Participants

Only human studies will be included in our review, and animal or cell studies will be excluded. Studies from all age groups or involving patients with any immune status are eligible.

Exposure

We have two exposures of interest for each research question in our review. For the first research question, the exposures are deficient or insufficient serum vitamin D levels in the participants. We will include articles in which the serum vitamin D levels are

For peer review only

identified as deficient or insufficient. Notably, no consensus exists about serum vitamin D levels, and different studies may use different definitions (Table 2). If eligible studies provide original values for the serum 25(OH)D levels, we will define vitamin D deficiency as 25(OH)D < 25 nmol/L and insufficiency as 25(OH)D in the range from 25-50 nmol/L in accordance with the standards of Public Health England[10].

For the second research question, the intervention is oral vitamin D supplementation, or oral vitamin D analogue treatment used for secondary hyperparathyroidism or osteoporosis (Supplementary Table 1). We will exclude studies using topical vitamin D analogues, because their effects on systemic serum vitamin D levels are unknown.

Comparators

The comparator groups for vitamin D insufficiency and deficiency are those with sufficient serum vitamin D levels. For those receiving oral vitamin D supplementation or treatment, the comparators are those without vitamin D supplementation or treatment, respectively.

Outcomes

The outcomes will be infection with or reactivation of all eight human herpesviruses listed in Table 1. Infection with and reactivation of herpesviruses are defined using clinical or laboratory criteria. The clinical criteria include patients presenting with classical symptoms, for instance, such as the painful rash of herpes zoster, or a diagnosis recorded by physicians. Laboratory criteria include using laboratory techniques to confirm the diagnosis, such as evaluation of the serum viral load by PCR. Studies reporting only serum antibodies against herpesviruses will not be included.

For peer review only

Information sources

We will search the following database from inception until June 2019: Medline (Ovid), EMBASE (Ovid), Web of Science, Scopus, Cochrane Library, and Global Health (Ovid). Grey literature databases, including Open Grey, Prevention Information & Evidence eLibrary, BASE, and EThOS, will also be searched. Finally, the clinical trial register at ClinicalTrials.gov will be searched to ensure the completeness of the literature.

Search strategy

We will search for synonyms of “human herpesviruses” and “vitamin D” using both controlled vocabularies and keywords in each database. A search strategy in Medline (Ovid) is listed in Supplementary Table 2. The subject headings will be modified for different databases. The results will be combined using the Boolean logic operator “AND.” For some database with limited search functions, such as single line search, keywords will be split into small sections to fulfil the requirement (Supplementary Table 3). In addition to searching electronic databases, we will manually search for the references lists of the included articles and relevant systematic reviews.

Study records

Data management

One researcher will import the search results from different databases into the citation management software EndNote X9 (Clarivate Analytics, version 9.1/2019). Duplicated results will be identified and deleted.

For peer review only

Selection process

Two researchers will independently screen the titles and abstracts of all identified studies. We will obtain full texts of all studies fulfilling the review criteria, and the two researchers will screen all articles to establish their eligibility for inclusion. Any discrepancy in the reviewing process will be adjudicated by the third researcher.

Data collection process

Data extraction for the first three studies will be performed by two independent researchers to ensure the integrity of the process; then, one researcher will extract data from the remaining studies. If any data are missing or unclear, we will contact the authors for further clarification and information.

Data items

We will summarize and extract data from the included studies using a Population, Exposure/Intervention, Comparator, Outcomes, and Study characteristics framework as follows:

1. Population: sample sizes of each study, inclusion or exclusion criteria for the participants, and demographic characteristics, such as sex, age, or immune status
2. Exposure 1: insufficient or deficit serum vitamin D levels of the study participants
   Exposure 2: oral vitamin D supplementation or vitamin D analogue
3. Comparator 1: sufficient serum vitamin D levels among the participants
   Comparator 2: without vitamin D supplementation or vitamin D analogue use

For peer review only

4. Outcomes: clinically diagnosed herpesvirus infection/reactivation or laboratory-confirmed herpesvirus infections
5. Study characteristics: publication details (authors, publication year, and journal), study designs, and study results

Outcomes and prioritization

The outcomes of the studies are herpesvirus infections or reactivation. The disease can be diagnosed either clinically or through laboratory confirmation. Some herpesvirus infections, such as herpes zoster, may lead to characteristic clinical symptoms, and some laboratory approaches, such as PCR, can detect the viral load, which can also be proof of herpesvirus infection/reactivation. Studies measuring serum anti-herpesvirus antibodies will not be included, because IgG antibodies detected in the absence of clinical symptoms cannot ascertain the timing of infection.

Risk of bias in individual studies

We will assess the risk of bias from the included studies using a template form based on the Cochrane approach for trials and observational studies, and all relevant domains of bias will be assessed for different study types. For randomized controlled trials, we will evaluate bias due to the following sources: 1. random sequence generation; 2. allocation concealment; 3. blinding of participants, personnel, or assessment; 4. incomplete outcome data; and 5. selective reporting. For observational studies, we will consider bias due to the following sources: 1.confounding factors; 2. selection of participants or controls; 3. differential and non-differential misclassification of the exposure or outcome; 4. reverse causation; and 5. missing data (Supplementary Table

For peer review only

4). A piloted form will be tested by applying it to extract data from the first three studies. To ensure the quality and consistency of the risk of bias assessment, the first three studies will be evaluated by two independent researchers. Then, one researcher will complete the evaluation of the remaining included studies. Any discrepancies will be examined by the third researcher.

Data synthesis and meta-bias (es)

We will comprehensively search different databases including grey literature databases to minimize bias in our search. We will use a narrative synthesis to summarize the data and results from the eligible studies included in our review. Since each herpesvirus subtypes have differing pathogenic pathways, we will display the results separately by virus. If an adequate numbers of studies with acceptable homogeneity are included, a meta-analysis will be performed to integrate the study results. We will decide whether to use fixed- or random-effects models by considering the I^2 value for heterogeneity; values >50% will be considered to represent substantial heterogeneity[11]. If the number of included studies is sufficient, we will carry out subgroup analyses of subjects with different ages, immune statuses, or latitude to explore the source of heterogeneity. A funnel plot will be used to present the distribution of studies and examine any possible publication bias[12]. All statistical analyses will be performed by using STATA version 15.

Confidence in cumulative evidence

We will evaluate the quality of evidence using the Grading of Recommendations, Assessment, Development and Evaluations (GRADE) framework[13]. We will evaluate the design and risk of bias across all included studies. Studies with significant effects,

For peer review only

strong dose responses, or that are well adjusted for plausible confounders will receive higher grades, whereas those with a higher risk of bias, inconsistency, indirectness, or imprecision will be downgraded. We will conclude the quality of evidence using a ‘Summary of Findings’ table and assign each outcome a quality rank of “high”, “moderate”, “low” or “very low” for the level of confidence.

ETHICS AND DISSEMINATION

Any changes in the study protocol will be recorded and reported. Any revision of the protocol will be recorded in the supplementation of the final published review.

For peer review only

AUTHORS' CONTRIBUTION

LYL contributed to the design of the study, drafted the introduction, methods and analysis and revised the protocol according to other authors' comments; CWG contributed to the design of the study, made critical comments on the protocol and revised the paper critically; SML contributed to the design of the study, made critical comments on the protocol and revised the paper critically; KB contributed to the design of the study and revised the paper critically; HJF contributed to the design of the study and revised the paper critically; LS contributed to the design of the study and revised the paper critically. All authors approved the final version of the protocol.

For peer review only

Table 1: List of human herpesviruses

Common Name	Abbreviation
Herpes simplex virus type 1 (HSV-1)	HHV-1
Herpes simplex virus type 2 (HSV-2)	HHV-2
Varicella-zoster virus (VZV)	HHV-3
Epstein-Barr virus (EBV)	HHV-4
Cytomegalovirus (CMV)	HHV-5
HHV-6 variant A	HHV-6A
HHV-6 variant B	HHV-6B
Kaposi's sarcoma–associated HV (KSHV)	HHV-8

For peer review only

Table 2: The normal range of serum vitamin D levels adapted in different studies

Study	Deficiency (nmol/L)	Insufficiency (nmol/L)	Adequate (nmol/L)
Pearce and Cheetham, 2010[14]	<25	25-50	50-75
Institute of Medicine, 2011[15]	<30	30-50	≥50
Holick et al., 2011[16]	<50	52.5-72.5	
Hanley et al., 2010[17]	<25	25-75	

* 1 nmol/L = 0.4 ng/mL

For peer review only

SUPPORT

This research received no specific grant from any funding agency in the public, commercial or not-for-profit sectors.

FUNDINF STATEMENT

LYL is funded by the scholarship of government sponsorship for overseas study by the Ministry of Education Republic of China (Taiwan). CWG is supported by a Wellcome Intermediate Clinical Fellowship (201440_Z_16_Z). SML is funded by a Wellcome Senior Clinical Fellowship in Science (205039/Z/16/Z). KB is supported by a National Institute of Health Research (NIHR) Doctoral Research Fellowship (DRF-2018-11-ST2-066).

COMPETING INTERESTS STATEMENT:

The authors declare that they have no known conflicts of interest.

For peer review only

REFERENCE:

1. Knipe DM, Howley P. Fields Virology: Wolters Kluwer Health 2013:1806.
2. Liesegang TJ. Herpes simplex virus epidemiology and ocular importance. *Cornea* 2001;20(1):1-13. [published Online First: 2001/02/24]
3. Young LS, Yap LF, Murray PG. Epstein-Barr virus: more than 50 years old and still providing surprises. *Nat Rev Cancer* 2016;16(12):789-802. doi: 10.1038/nrc.2016.92
4. Gauthier A, Breuer J, Carrington D, et al. Epidemiology and cost of herpes zoster and post-herpetic neuralgia in the United Kingdom. *Epidemiology and infection* 2009;137(1):38-47. doi: 10.1017/s0950268808000678 [published Online First: 2008/05/10]
5. Holick MF. Vitamin D deficiency. *The New England journal of medicine* 2007;357(3):266-81. doi: 10.1056/NEJMra070553 [published Online First: 2007/07/20]
6. Ginsberg J, Mohebbi MH, Patel RS, et al. Detecting influenza epidemics using search engine query data. *Nature* 2009;457(7232):1012-4. doi: 10.1038/nature07634 [published Online First: 2008/11/21]
7. Su G, Liu Z, Qin X, et al. Vitamin D deficiency and treatment versus risk of infection in end-stage renal disease patients under dialysis: a systematic review and meta-analysis. *Nephrol Dial Transplant* 2018 doi: 10.1093/ndt/gfy216 [published Online First: 2018/07/31]
8. Martineau AR, Jolliffe DA, Hooper RL, et al. Vitamin D supplementation to prevent acute respiratory tract infections: systematic review and meta-analysis of individual participant data. *Bmj* 2017;356:i6583. doi: https://dx.doi.org/10.1136/bmj.i6583
9. Shamseer L, Moher D, Clarke M, et al. Preferred reporting items for systematic review and meta-analysis protocols (PRISMA-P) 2015: elaboration and explanation. 2015;349:g7647. doi: 10.1136/bmj.g7647 %J BMJ : British Medical Journal
10. The Scientific Advisory Committee on Nutrition. Vitamin D and Health: Public Health England 2016.
11. Jonathan J Deeks, Julian PT Higgins, Douglas G Altman. 9.5.2 Identifying and measuring heterogeneity. In: Douglas G Altman, ed. Cochrane Handbook for Systematic Reviews of Intervention Version 510 (updated March 2011): The Cochrane Collaboration 2011.
12. Jonathan AC Sterne, Matthias Egger, David Moher. 10.4.1 Funnel plots. In: Moher D, ed. Cochrane Handbook for Systematic Reviews of Intervention Version 510 (updated March 2011): The Cochrane Collaboration 2011.
13. Guyatt GH, Oxman AD, Vist GE, et al. GRADE: an emerging consensus on rating quality of evidence and strength of recommendations. *Bmj* 2008;336(7650):924-26. doi: 10.1136/bmj.39489.470347.AD
14. Pearce SH, Cheetham TD. Diagnosis and management of vitamin D deficiency. 2010;340:b5664. doi: 10.1136/bmj.b5664 %J BMJ

For peer review only

15. Institute of Medicine. Dietary Reference Intakes for Calcium and Vitamin D. Washington (DC): National Academies Press (US) 2011.
16. Holick MF, Binkley NC, Bischoff-Ferrari HA, et al. Evaluation, treatment, and prevention of vitamin D deficiency: an Endocrine Society clinical practice guideline. *The Journal of clinical endocrinology and metabolism* 2011;96(7):1911-30. doi: 10.1210/jc.2011-0385 [published Online First: 2011/06/08]
17. Hanley DA, Cranney A, Jones G, et al. Vitamin D in adult health and disease: a review and guideline statement from Osteoporosis Canada (summary). *Cmaj* 2010;182(12):1315-9. doi: 10.1503/cmaj.091062 [published Online First: 2010/07/14]

For peer review only

Supplementary Table 1: List of oral vitamin D analogues

Name	Indication	Brand name
Paricalcitol (19-nor-1α,25(OH)2D2)	Secondary hyperparathyroidism	Zemplar® (Abbott)
Doxercalciferol (1α(OH)D2)	Secondary hyperparathyroidism	Hectorol® (Genzyme corp)
Falecalcitriol (26,27 F6-1α,25(OH)2D3)	Secondary hyperparathyroidism (Japan only)	Fulstan® (Dainippon Sumitomo) and Hornel® (Taisho Yakuhin)
Maxacalcitol (22oxa-1α,25(OH)2D3)	Secondary hyperparathyroidism and psoriasis (Japan only)	Oxarol® (Chugai Pharmaceutical)
Eldecalcitol (2β-(3-hydroxypropoxy)-1α,25(OH)2D3)	Osteoporosis (Japan only)	Edirol® (Chugai Pharmaceutical)

For peer review only

Supplementary Table 2: Search strategies in Medline (Ovid)

Item	Search Terms
Herpesviruses	
1	(HHV adj1 ("1" or "2" or "3" or "4" or "5" or "6" or "7" or "8")) or (HHV?1 or HHV?2 or HHV?3 or HHV?4 or HHV?5 or HHV?6 or HHV?7 or HHV?8)
2	exp Herpesviridae Infections/ or exp Herpesviridae/
3	herpes* or herpesvir*
4	exp Herpes Simplex/
5	herpes simplex
6	HSV-1 or HSV?1
7	Cold sore*
8	exp Herpes Labialis/
9	Herpes Labialis
10	Herpes* adj2 Labial*
11	Hsv-2 or HSV?2 or (genit* herpes* or genit* sores)
12	exp Herpes Zoster/
13	herpes zoster
14	vz virus* or VZV
15	varicella?
16	chickenpox or Chicken?pox
17	shingle*
18	neuralgia adj1 post?herpetic)
19	Epstein-Barr virus* or (burkitt lymphoma adj2 virus*) or e?b virus* or ((eb or e-b) adj virus*) or ebv or Epstein?barr virus* or infectious mononucleosis or glandular fever
20	Cytomegalovirus* or CMV
21	salivary gland virus* or hblv or betaherpesvirus or b-lymphotropic virus* or cihhv or icihhv or Roseolovirus or Roseola Infantum or Exanthema Subitum
22	Kaposi* sarcoma-associated herpesvir* or KSHV or Primary Effusion Lymphoma
23	ac?clovir or Zovirax or valac?clovir or valtrex or famc?clovir or famvir or penc?clovir or ganc?clovir or cidofovir or foscarnet* or valganc?clovir or lubocavir or brivudin or Docosanol or Sorivudine or Idoxuridine or Trifluridine
24	1 or 2 or 3 or 4 or 5 or 6 or 7 or 8 or 9 or 10 or 11 or 12 or 13 or 14 or 15 or 16 or 17 or 18 or 19 or 20 or 21 or 22 or 23
Vitamin D	
25	exp Vitamin D/ or exp Vitamin D Deficiency/
26	(vitamin* or hydroxyvitamin* or dihydroxyvitamin*) adj1 d*
27	c?olecalciferol* or calciol* or calcitriol* or ergocalciferol* or calcifediol* or calcid?ol* or calciferol* or ercalcidiol* or ercalcitriol* or hydroxyergocalciferol* or hydroxycalciferol* or hydroxyc?olecalciferol* or dihydroxyc?olecalciferol* or ergosterol
28	(dihydrotachyst* or alphacalcid?ol or alfacalcid?ol or Falecalcitriol* or Maxacalcitol* or Oxacalcitriol* or Paricalcitol* or Lexacalcitol* or Seocalcitol* or Calcipotriol* or vitamin D analogue*
29	exp Familial Hypophosphatemic Rickets/ or exp Rickets, Hypophosphatemic/ or exp Rickets/
30	Rickets
31	rachitides
32	rachitis
33	25 or 26 or 27 or 28 or 29 or 30 or 31 or 32
34	24 and 33

For peer review only

Supplementary Table 3: modified keywords for grey literature search

Search Concept	Keywords Groups
Herpesviruses	Group 1: herpesvir* OR herpes* OR HHV* OR HSV* OR Cold sore* OR varicella* OR shingle*<br>Group 2: chickenpox OR post-herpetic neuralgia OR Burkitt lymphoma OR EBV OR Epstein-Barr<br>Group 3: Cytomegalovirus* or CMV OR Kaposi* sarcoma-associated herpesvir* or KSHV OR famc*
Vitamin D	Group 4: "vitamin D" or hydroxyvitamin* or dihydroxyvitamin*<br>Group 5: calcitriol* OR cholecalciferol OR ergocalciferol* OR Calcidol<br>Group 6: calcifer* OR ergosterol OR rickets OR Calcipotriol* OR vitamin D analogue
Combination of concepts	(Group 1, Group 2, Group 3) x (Group 4, Group 5, Group 6)

For peer review only

Supplementary Table 4: Domains of risk of bias assessment

Domains	Explanation
Confounders	Not adjusted for confounders
Selection bias	Intervention studies: random sequence generation and allocation concealment<br>Observational studies: and participation bias, selection of participants, and selection of controls
Misclassification of exposure and outcomes	Differential: recall bias, observer bias<br>Non-differential: ascertainment bias
Performance bias and detection bias	Blinding of participants, personnel and outcome assessment
Attrition bias	Incomplete outcome data
Bias due to Missing Data	Differential loss to follow up and exclusion of individuals with missing data
Reporting bias	Selective reporting
Reverse causation	Defined outcomes prior to the exposures

For peer review only

Reporting checklist for protocol of a systematic review.

Based on the PRISMA-P guidelines.

Instructions to authors

Complete this checklist by entering the page numbers from your manuscript where readers will find each of the items listed below.

Your article may not currently address all the items on the checklist. Please modify your text to include the missing information. If you are certain that an item does not apply, please write "n/a" and provide a short explanation.

Upload your completed checklist as an extra file when you submit to a journal.

In your methods section, say that you used the PRISMA-Preporting guidelines, and cite them as:

Moher D, Shamseer L, Clarke M, Ghersi D, Liberati A, Petticrew M, Shekelle P, Stewart LA. Preferred Reporting Items for Systematic Review and Meta-Analysis Protocols (PRISMA-P) 2015 statement. Syst Rev. 2015;4(1):1.

		Reporting Item	Page Number
**Title**			
Identification	#1a	Identify the report as a protocol of a systematic review	1
Update	#1b	If the protocol is for an update of a previous systematic review, identify as such	n/a

For peer review only

**Registration**			
	#2	If registered, provide the name of the registry (such as PROSPERO) and registration number	4
**Authors**			
Contact	#3a	Provide name, institutional affiliation, e-mail address of all protocol authors; provide physical mailing address of corresponding author	1
Contribution	#3b	Describe contributions of protocol authors and identify the guarantor of the review	15
**Amendments**			
	#4	If the protocol represents an amendment of a previously completed or published protocol, identify as such and list changes; otherwise, state plan for documenting important protocol amendments	n/a
**Support**			
Sources	#5a	Indicate sources of financial or other support for the review	18
Sponsor	#5b	Provide name for the review funder and / or sponsor	18
Role of sponsor or funder	#5c	Describe roles of funder(s), sponsor(s), and / or institution(s), if any, in developing the protocol	n/a
**Introduction**			
Rationale	#6	Describe the rationale for the review in the context of what is	6

For peer review only

		already known	
Objectives	#7	Provide an explicit statement of the question(s) the review will address with reference to participants, interventions, comparators, and outcomes (PICO)	7
**Methods**			
Eligibility criteria	#8	Specify the study characteristics (such as PICO, study design, setting, time frame) and report characteristics (such as years considered, language, publication status) to be used as criteria for eligibility for the review	8
Information sources	#9	Describe all intended information sources (such as electronic databases, contact with study authors, trial registers or other grey literature sources) with planned dates of coverage	10
Search strategy	#10	Present draft of search strategy to be used for at least one electronic database, including planned limits, such that it could be repeated	10
Study records - data management	#11a	Describe the mechanism(s) that will be used to manage records and data throughout the review	10
Study records - selection process	#11b	State the process that will be used for selecting studies (such as two independent reviewers) through each phase of the review (that is, screening, eligibility and inclusion in meta-analysis)	11
Study records - data collection	#11c	Describe planned method of extracting data from reports (such as piloting forms, done independently, in duplicate), any	11

For peer review only

process		processes for obtaining and confirming data from investigators	
Data items	#12	List and define all variables for which data will be sought (such as PICO items, funding sources), any pre-planned data assumptions and simplifications	11
Outcomes and prioritization	#13	List and define all outcomes for which data will be sought, including prioritization of main and additional outcomes, with rationale	12
Risk of bias in individual studies	#14	Describe anticipated methods for assessing risk of bias of individual studies, including whether this will be done at the outcome or study level, or both; state how this information will be used in data synthesis	12
Data synthesis	#15a	Describe criteria under which study data will be quantitatively synthesised	13
Data synthesis	#15b	If data are appropriate for quantitative synthesis, describe planned summary measures, methods of handling data and methods of combining data from studies, including any planned exploration of consistency (such as I2, Kendall's τ)	13
Data synthesis	#15c	Describe any proposed additional analyses (such as sensitivity or subgroup analyses, meta-regression)	13
Data synthesis	#15d	If quantitative synthesis is not appropriate, describe the type of summary planned	13
Meta-bias(es)	#16	Specify any planned assessment of meta-bias(es) (such as publication bias across studies, selective reporting within	13

For peer review only

		studies)	
Confidence in cumulative evidence	#17	Describe how the strength of the body of evidence will be assessed (such as GRADE)	13

The PRISMA-P checklist is distributed under the terms of the Creative Commons Attribution License CC-BY 4.0. This checklist was completed on 22. May 2019 using https://www.goodreports.org/, a tool made by the EQUATOR Network in collaboration with Penelope.ai

For peer review only

BMJ Open

Vitamin D deficiency or supplementation and the risk of human herpesvirus infections or reactivation: a systematic review protocol

Journal:	*BMJ Open*
Manuscript ID	bmjopen-2019-031867.R1
Article Type:	Protocol
Date Submitted by the Author:	06-Aug-2019
Complete List of Authors:	Lin, Liang-Yu; London School of Hygiene and Tropical Medicine Faculty of Epidemiology and Population Health, Department of Non-communicable Disease Epidemiology<br>Bhate, Ketaki; London School of Hygiene and Tropical Medicine Faculty of Epidemiology and Population Health, Department of Non-communicable Disease Epidemiology<br>Forbes, Harriet; London School of Hygiene and Tropical Medicine Faculty of Epidemiology and Population Health, Department of Non-communicable Disease Epidemiology<br>Smeeth, Liam; London School of Hygiene and Tropical Medicine Faculty of Epidemiology and Population Health, Department of Non-communicable Disease Epidemiology<br>Warren-Gash, Charlotte; London School of Hygiene and Tropical Medicine Faculty of Epidemiology and Population Health, Department of Non-communicable Disease Epidemiology<br>Langan, Sinead; London School of Hygiene and Tropical Medicine Faculty of Epidemiology and Population Health, Department of Non-communicable Disease Epidemiology
**Primary Subject Heading**:	Infectious diseases
Secondary Subject Heading:	Nutrition and metabolism
Keywords:	systematic review, herpesviridae, vitamin D deficiency, vitamin D supplementation

SCHOLARONE™
Manuscripts

Title: Vitamin D deficiency or supplementation and the risk of human herpesvirus infections or reactivation: a systematic review protocol

Authors Contact

Dr Liang-Yu Lin[1]. LSHTM liang-yu.lin@lshtm.ac.uk ORCID: https://orcid.org/0000-0003-4720-6738

Dr Ketaki Bhate[1]. LSHTM Ketaki.Bhate@lshtm.ac.uk

ORCID: https://orcid.org/0000-0001-5509-4428

Dr Harriet Forbes[1]. LSHTM Harriet.Forbes@lshtm.ac.uk

ORCID: https://orcid.org/0000-0001-6888-2212

Professor Liam Smeeth[1]. LSHTM Liam.Smeeth@lshtm.ac.uk

ORCID: https://orcid.org/0000-0002-9168-6022

Dr Charlotte Warren-Gash[1]*. LSHTM Charlotte.Warren-Gash1@lshtm.ac.uk

ORCID: https://orcid.org/0000-0003-4524-3180

Professor Sinéad M Langan[1]*. LSHTM Sinead.Langan@LSHTM.ac.uk

ORCID: https://orcid.org/0000-0002-7022-7441

* The senior authors contributed equally to this work

Authors' Affiliations:

1. Faculty of Epidemiology & Population Health, London School of Hygiene and Tropical Medicine, London, UK

For peer review only

Correspondence to: Professor Sinéad M Langan

LSHTM, Keppel Street, London, WC1E 7HT, United Kingdom

Tel: 0207 9272680, Sinead.Langan@LSHTM.ac.uk

Word count: 2672

For peer review only

Keywords: systematic review, herpesviridae, vitamin D deficiency, vitamin D supplementation

ABSTRACT:

Introduction:

Human herpesviruses induce lifelong latent infections and may reactivate as the immune system deteriorates. Recent studies have suggested that vitamin D, an essential element of bone health, may have some effect of protecting against infections, but investigations of its potential to prevent herpesvirus infection or reactivation are limited. We will review the current literature examining vitamin D and the risk of herpesvirus infections or reactivation.

Methods and analysis:

Our systematic review will address two research questions: 1. Do deficient/insufficient serum vitamin D levels increase the risk of herpesvirus infections, and 2. Does vitamin D supplementation protect against herpesvirus infections? We will include only intervention studies with control groups, cohort studies and case-control studies. We will use subject headings and keywords to search for synonyms of "vitamin D" and "herpesviruses" (including herpes simplex virus type 1 and 2, varicella-zoster virus, cytomegalovirus, Epstein-Barr virus and human herpesviruses type 6,7 and 8) in Medline, Embase, Global Health, Web of Science, Scopus, and Cochrane Central Register of Controlled Trials, and the grey literature databases Prevention Information & Evidence eLibrary, Open Grey, EThOS, and BASE from inception to 31 August 2019. References to the included articles and relevant systematic reviews will also be examined. Two reviewers will independently screen the study titles and abstracts, and examine the full texts to decide the final eligibility. They will independently extract data

For peer review only

from the studies and assess bias using the Cochrane Collaboration approach. A third researcher will solve any discrepancies. The results will be narratively synthesized; if an adequate number of studies is included and the homogeneity between studies is acceptable, a meta-analysis will be performed. We will assess the quality of evidence using the Grading of Recommendations, Assessment, Development and Evaluation (GRADE) framework, and display the results in a summary of findings table.

Ethics and dissemination

Ethical review is not required for a systematic review. We will publish the results in a peer-review journal. Any amendments to the protocol will be recorded in the supplementary section.

PROSPERO registration number:

CRD42019130153

For peer review only

STRENGTHS AND LIMITATION OF THIS STUDY:

- This systematic review will be undertaken following the predefined population, exposure, comparator and outcome framework.
- All available databases, including six major databases and four grey literature databases, will be searched to obtain all eligible studies.
- The summarized results will improve our understanding of the current evidence of the possible association between vitamin D and herpesvirus infection/reactivation.
- The number of sufficient eligible studies may be inadequate, especially for some viruses that are harder to diagnose; also, the included studies may not have an adequate quality of evidence to answer the research questions.

For peer review only

INTRODUCTION

Rationale

Herpesviruses are a group of double-stranded DNA viruses that infect humans and some animals. After infecting their hosts, these viruses cannot be eradicated; instead, they establish latency and persist for life. As the host's immunity declines, these viruses can reactivate to induce various symptoms. There are nine human herpesviruses (Table 1).[1] Reactivation of herpesviruses may induce serious complications. For instance, herpes simplex viruses 1 (HSV-1) can lead to herpetic keratitis, which is the major cause of blindness in developed countries;[2] Epstein-Barr virus (EBV) may induce nasopharyngeal cancer;[3] and Varicella zoster virus (VZV) would cause herpes zoster and post-herpetic neuralgia, which increases financial burdens especially for people older than aged 65 years.[4] Consequently, investigating immunomodulatory factors associated with infection or reactivation of this virus family is important.

Vitamin D is mainly endogenously synthesized by the skin after sun exposure and can be supplied through dietary intake and supplementation. It plays an important role in absorbing calcium and phosphate, which are essential for bone health.[5] Recently, some studies have indicated that vitamin D may have potential immunomodulatory effects associated with the regulation of antimicrobial peptides (AMPs).[6] In previous cell studies, vitamin D induced gene expression of an AMP named Cathelicidin. In response to pathogen exposure, immune cells such as monocytes or macrophages, upregulate vitamin D receptors and enzymes to increase the production of Cathelicidin.[7-9] In addition, evidence suggests that vitamin D has some effects on the adaptive immune

For peer review only

system. Vitamin D suppresses CD4+ Th 1 lymphocytes and increases Th 2 lymphocytes, and it also intensifies the effect of regulatory T lymphocyte responses.[10 11] Regarding the effects of vitamin D associated AMPs on herpesviruses, a cell study indicated that Cathelicidin decreased HSV-1 viral titres isolated from keratoconjunctivitis patients;[12] furthermore, another cell study also showed that vitamin D supplementation reduced HSV-1 viral load and mRNA expression in HSV-1 infected cells.[13]

Vitamin D also shows some anti-infective potential in epidemiological studies. A meta-analysis using original patient data from 25 randomized controlled trials showed that among the general population, vitamin D supplementation reduced the risk of acute respiratory infections.[14] Furthermore, there is some evidence to suggest an anti-infective effect of vitamin D in specific patient groups, such as patients with chronic kidney disease (CKD), HIV or hepatitis C virus (HCV) infection. Among CKD patients receiving dialysis, a case-control study indicated that the risk of herpes zoster reactivation was significantly lower in those who received vitamin D supplementation;[15] another meta-analysis also showed that CKD patients with higher or normal serum vitamin D levels had a lower risk of infection.[16] Among HIV infected patients, lower serum vitamin D levels were also associated with a higher risk of clinical progression to AIDS and all-cause mortality in a cohort study,[17] while vitamin D supplementation didn't affect mortality, CD4 cell count or viral load.[18] For HCV infected patients, serum vitamin D levels were inversely associated with the grade of liver inflammation and the stage of fibrosis,[19] while no protective effect of vitamin D supplementation was seen in a meta-analysis of clinical trials.[20] However, the effect of vitamin D on herpesvirus infection or reactivation in the general population is unclear.

For peer review only

In this study, we will comprehensively review studies of the effect of serum vitamin D levels or the use of oral vitamin D supplementation on infection with or reactivation of any of the eight human herpesviruses.

OBJECTIVE

This review aims to explore the association between vitamin D and herpesviruses. The proposed systematic review will address two primary research questions:

1. Is serum vitamin D deficiency/insufficiency associated with an increased risk of infection with or reactivation of human herpesviruses?
2. Does oral vitamin D supplementation protect against infection with or reactivation of human herpesviruses?

For peer review only

METHODS

This study protocol will be reported according to the Preferred Reporting Items for Systematic Review and Meta-Analysis Protocols (PRISMA-P).[21]

Patient and Public Involvement

Patients and or the public were not involved in this systematic review protocol.

Eligibility Criteria

Study Design and Characteristics

To identify the possible causal association between vitamin D and herpesvirus infections, we will review observational studies, including cohort and case-control studies, and intervention studies with any type of control group, either placebo, active comparator or no treatment, including randomized or non-randomized controlled trials. Descriptive studies, ecological studies, cross-sectional studies, case reports and case series will not be included. If a systematic review relevant to our topic is found, we will review its references.

Participants

Only human studies will be included in our review, and animal or cell studies will be excluded. Studies from all age groups or involving patients with any immune status are eligible.

For peer review only

Exposure

We have two exposures of interest for each research question in our review. For the first research question, the exposures are deficient or insufficient serum vitamin D levels in the participants. We will include articles in which the serum vitamin D levels are identified as deficient or insufficient. Notably, no consensus exists about serum vitamin D levels, and different studies may use different definitions (Table 2). If eligible studies provide original values for the serum 25(OH)D levels, we will define vitamin D deficiency as 25(OH)D < 25 nmol/L and insufficiency as 25(OH)D in the range from 25-50 nmol/L in accordance with the standards of Public Health England[22].

For the second research question, the intervention is oral vitamin D supplementation or oral vitamin D analogue treatment used for secondary hyperparathyroidism or osteoporosis (Supplementary Table 1). We will exclude studies using topical vitamin D analogues, because their effects on systemic serum vitamin D levels are unknown.

Comparators

The comparator groups for vitamin D insufficiency and deficiency are those with sufficient serum vitamin D levels. For those receiving oral vitamin D supplementation or treatment, the comparators are those without vitamin D supplementation or treatment or receiving placebo or another active comparator, respectively.

Outcomes

The outcomes will be infection with or reactivation of all eight human herpesviruses listed in Table 1. Infection with and reactivation of herpesviruses are defined using

For peer review only

140 clinical or laboratory criteria. The clinical criteria include patients presenting with classical symptoms, for instance, such as the painful rash of herpes zoster, or a diagnosis recorded by physicians. Laboratory criteria include using laboratory techniques to confirm the diagnosis, such as evaluation of the serum viral load by PCR. Studies reporting only serum antibodies against herpesviruses will not be included.

Information sources

We will search the following database from inception to 31 August 2019: Medline (Ovid), EMBASE (Ovid), Web of Science, Scopus, Cochrane Library, and Global Health (Ovid). To enhance the sensitivity of our search and reduce the risk of publication bias, we will also search grey literature databases such as Open Grey, Prevention Information & Evidence eLibrary, BASE, EThOS and the clinical trials register at ClinicalTrials.gov.

Search strategy

We will search for synonyms of “human herpesviruses” and “vitamin D” using both controlled vocabularies and keywords in each database. A search strategy in Medline (Ovid) is listed in Supplementary Table 2. The subject headings will be modified for different databases. The results will be combined using the Boolean logic operator “AND.” For some database with limited search functions, such as single line search, keywords will be split into small sections to fulfil the requirement (Supplementary Table 3). In addition to searching electronic databases, we will manually search for the references lists of the included articles and relevant systematic reviews.

For peer review only

Study records

Data management

One researcher will import the search results from different databases into the citation management software EndNote X9 (Clarivate Analytics, version 9.1/2019). Duplicated results will be identified and deleted.

Selection process

Two researchers will independently screen the titles and abstracts of all identified studies. We will obtain full texts of all studies fulfilling the review criteria, and the two researchers will screen all articles to establish their eligibility for inclusion. Any discrepancy in the reviewing process will be adjudicated by the third researcher.

Data collection process

Data extraction for the first three studies will be performed by two independent researchers to ensure the integrity of the process; then, one researcher will extract data from the remaining studies. If any data are missing or unclear, we will contact the authors for further clarification and information.

Data items

We will summarize and extract data from the included studies using a Population, Exposure/Intervention, Comparator, Outcomes, and Study characteristics framework as follows:

For peer review only

1. Population: sample sizes of each study, inclusion or exclusion criteria for the participants, and demographic characteristics, such as sex, age, or immune status
2. Exposure 1: insufficient or deficit serum vitamin D levels of the study participants
   Exposure 2: oral vitamin D supplementation or vitamin D analogue
3. Comparator 1: sufficient serum vitamin D levels among the participants
   Comparator 2: without vitamin D supplementation or vitamin D analogue use
4. Outcomes: definition of herpesvirus infection/reactivation, i.e. clinically diagnosed or laboratory-confirmed herpesvirus infections; the number of study subjects with the outcomes
5. Study characteristics: publication details (authors, publication year, country, and journal), study designs, confounders measured, confounders adjusted and study results

Outcomes and prioritization

The outcomes of the studies are herpesvirus infections or reactivation. The disease can be diagnosed either clinically or through laboratory confirmation. Some herpesvirus infections, such as herpes zoster, may lead to characteristic clinical symptoms, and some laboratory approaches, such as PCR, can detect the viral load, which can also be proof of herpesvirus infection/reactivation. Studies measuring serum anti-herpesvirus antibodies will not be included, because IgG antibodies detected in the absence of clinical symptoms cannot ascertain the timing of infection. Regarding the study results, our focus is on the incidence of herpesviruses infection or reactivation. We will report the outcome definition used for each study, and will extract data on the appropriate

For peer review only

effect measure. The effect measures will include odds ratios for case-control studies and risk, rate or hazard ratios for cohort studies or clinical trials. If studies report only continuous outcomes such as viral load, then we will summarise these using means or medians as appropriate.

Risk of bias in individual studies

We will assess the risk of bias from the included studies using a template form based on the Cochrane approach for trials and observational studies, and all relevant domains of bias will be assessed for different study types. For randomized controlled trials, we will evaluate bias due to the following sources: 1. random sequence generation; 2. allocation concealment; 3. blinding of participants, personnel, or assessment; 4. incomplete outcome data; and 5. selective reporting. For observational studies, we will consider bias due to the following sources: 1. confounding factors; 2. selection of participants or controls; 3. differential and non-differential misclassification of the exposure or outcome; 4. reverse causation; and 5. missing data (Supplementary Table 4). A piloted form will be tested by applying it to extract data from the first three studies. To ensure the quality and consistency of the risk of bias assessment, the first three studies will be evaluated by two independent researchers. Then, one researcher will complete the evaluation of the remaining included studies. Any discrepancies will be examined by the third researcher.

Data synthesis and meta-bias (es)

We will comprehensively search different databases including grey literature databases to minimize bias in our search. We will use a narrative synthesis to summarize the data

For peer review only

and results from the eligible studies included in our review. Since each herpesvirus subtypes have different pathogenic pathways, we will display the results separately by virus. If an adequate number of studies with acceptable homogeneity are included, a meta-analysis will be performed to integrate the study results. We will decide whether to use fixed- or random-effects models by considering the I^2 value for heterogeneity; values >50% will be considered to represent substantial heterogeneity[23]. If the number of included studies is sufficient, we will carry out subgroup analyses of subjects with baseline vitamin D status, intervention trials, study durations, different ages, immune statuses, or latitude to explore sources of heterogeneity. A funnel plot will be used to present the distribution of studies and examine any possible publication bias[24]. All statistical analyses will be performed by using STATA version 15.

Confidence in cumulative evidence

We will evaluate the quality of evidence using the Grading of Recommendations, Assessment, Development and Evaluations (GRADE) framework[25]. We will evaluate the design and risk of bias across all included studies. Studies with significant effects, strong dose responses, or that are well adjusted for plausible confounders will receive higher grades, whereas those with a higher risk of bias, inconsistency, indirectness, or imprecision will be downgraded. We will conclude the quality of evidence using a ‘Summary of Findings’ table and assign each outcome a quality rank of “high”, “moderate”, “low” or “very low” for the level of confidence.

For peer review only

Ethics and dissemination

Any changes in the study protocol will be recorded and reported. Any revision of the protocol will be recorded in the supplementation of the final published review.

For peer review only

AUTHORS' CONTRIBUTION

LYL contributed to the design of the study, drafted the introduction, methods and analysis and revised the protocol according to other authors' comments; CWG contributed to the design of the study, made critical comments on the protocol and revised the paper critically; SML contributed to the design of the study, made critical comments on the protocol and revised the paper critically; KB contributed to the design of the study and revised the paper critically; HJF contributed to the design of the study and revised the paper critically; LS contributed to the design of the study and revised the paper critically. All authors approved the final version of the protocol.

For peer review only

Table 1: List of human herpesviruses

Common Name	Abbreviation
Herpes simplex virus type 1 (HSV-1)	HHV-1
Herpes simplex virus type 2 (HSV-2)	HHV-2
Varicella-zoster virus (VZV)	HHV-3
Epstein-Barr virus (EBV)	HHV-4
Cytomegalovirus (CMV)	HHV-5
HHV-6 variant A	HHV-6A
HHV-6 variant B	HHV-6B
HHV-7	HHV-7
Kaposi's sarcoma–associated HV (KSHV)	HHV-8

For peer review only

Table 2: The normal range of serum vitamin D levels adapted in different studies

Study	Deficiency (nmol/L)	Insufficiency (nmol/L)	Adequate (nmol/L)
Pearce and Cheetham, 2010[26]	<25	25-50	50-75
Institute of Medicine, 2011[27]	<30	30-50	≥50
Holick et al., 2011[28]	<50	52.5-72.5	
Hanley et al., 2010[29]	<25	25-75	

* 1 nmol/L = 0.4 ng/mL

For peer review only

SUPPORT

This research received no specific grant from any funding agency in the public, commercial or not-for-profit sectors.

FUNDING STATEMENT

LYL is funded by the scholarship of government sponsorship for overseas study by the Ministry of Education Republic of China (Taiwan). CWG is supported by a Wellcome Intermediate Clinical Fellowship (201440_Z_16_Z). SML is funded by a Wellcome Senior Clinical Fellowship in Science (205039/Z/16/Z). KB is supported by a National Institute of Health Research (NIHR) Doctoral Research Fellowship (DRF-2018-11-ST2-066).

COMPETING INTERESTS STATEMENT:

The authors declare that they have no known conflicts of interest.

For peer review only

References:

1. Knipe DM, Howley P. Fields Virology: Wolters Kluwer Health 2013:1806.

2. Liesegang TJ. Herpes simplex virus epidemiology and ocular importance. *Cornea* 2001;20(1):1-13. [published Online First: 2001/02/24]

3. Young LS, Yap LF, Murray PG. Epstein-Barr virus: more than 50 years old and still providing surprises. *Nat Rev Cancer* 2016;16(12):789-802. doi: 10.1038/nrc.2016.92

4. Scott FT, Johnson RW, Leedham-Green M, et al. The burden of Herpes Zoster: a prospective population based study. *Vaccine* 2006;24(9):1308-14. doi: 10.1016/j.vaccine.2005.09.026 [published Online First: 2005/12/15]

5. Holick MF. Vitamin D deficiency. *The New England journal of medicine* 2007;357(3):266-81. doi: 10.1056/NEJMra070553 [published Online First: 2007/07/20]

6. Zasloff M. Antimicrobial peptides of multicellular organisms. *Nature* 2002;415(6870):389-95. doi: 10.1038/415389a [published Online First: 2002/01/25]

7. Gombart AF, Borregaard N, Koeffler HP. Human cathelicidin antimicrobial peptide (CAMP) gene is a direct target of the vitamin D receptor and is strongly up-regulated in myeloid cells by 1,25-dihydroxyvitamin D3. *Faseb J* 2005;19(9):1067-77. doi: 10.1096/fj.04-3284com [published Online First: 2005/06/30]

8. Wang TT, Nestel FP, Bourdeau V, et al. Cutting edge: 1,25-dihydroxyvitamin D3 is a direct inducer of antimicrobial peptide gene expression. *J Immunol*

For peer review only

2004;173(5):2909-12. doi: 10.4049/jimmunol.173.5.2909 [published Online First: 2004/08/24]

9. Weber G, Heilborn JD, Chamorro Jimenez CI, et al. Vitamin D induces the antimicrobial protein hCAP18 in human skin. *The Journal of investigative dermatology* 2005;124(5):1080-2. doi: 10.1111/j.0022-202X.2005.23687.x [published Online First: 2005/04/28]

10. Cantorna MT, Yu S, Bruce D. The paradoxical effects of vitamin D on type 1 mediated immunity. *Molecular aspects of medicine* 2008;29(6):369-75. doi: 10.1016/j.mam.2008.04.004 [published Online First: 2008/05/04]

11. Mann EH, Pfeffer PE, Hawrylowicz CM. Vitamin D and Adaptive Immunology in Health and Disease. Vitamin D. Fourth Edi ed: Elsevier, 2018:937-49.

12. Gordon YJ, Huang LC, Romanowski EG, et al. Human cathelicidin (LL-37), a multifunctional peptide, is expressed by ocular surface epithelia and has potent antibacterial and antiviral activity. *Current eye research* 2005;30(5):385-94. doi: 10.1080/02713680590934111 [published Online First: 2005/07/16]

13. Kumar A, Singh MP, Kumar RS, et al. 25-Hydroxyvitamin D3 and 1,25 Dihydroxyvitamin D3 as an Antiviral and Immunomodulator Against Herpes Simplex Virus-1 Infection in HeLa Cells. *Viral Immunol* 2018;31(8):589-93. doi: 10.1089/vim.2018.0026 [published Online First: 2018/05/25]

14. Martineau AR, Jolliffe DA, Hooper RL, et al. Vitamin D supplementation to prevent acute respiratory tract infections: systematic review and meta-analysis of individual participant data. *Bmj* 2017;356:i6583. doi: https://dx.doi.org/10.1136/bmj.i6583

For peer review only

15. Chao CT, Lai CF, Huang JW. Risk factors for herpes zoster reactivation in maintenance hemodialysis patients. *Eur* 2012;23(8):711-5. doi: https://dx.doi.org/10.1016/j.ejim.2012.08.005
16. Su G, Liu Z, Qin X, et al. Vitamin D deficiency and treatment versus risk of infection in end-stage renal disease patients under dialysis: a systematic review and meta-analysis. *Nephrol Dial Transplant* 2018 doi: 10.1093/ndt/gfy216 [published Online First: 2018/07/31]
17. Viard J-Pa, Souberbielle J-Cb, Kirk Oc, et al. Vitamin D and clinical disease progression in HIV infection: results from the EuroSIDA study. *Aids* 2011;25(10):1305-15.
18. Visser ME, Durao S, Sinclair D, et al. Micronutrient supplementation in adults with HIV infection. *Cochrane Database Syst Rev* 2017;5:CD003650. doi: https://dx.doi.org/10.1002/14651858.CD003650.pub4
19. Petta S, Camma C, Scazzone C, et al. Low vitamin D serum level is related to severe fibrosis and low responsiveness to interferon-based therapy in genotype 1 chronic hepatitis C. *Hepatology* 2010;51(4):1158-67. doi: 10.1002/hep.23489 [published Online First: 2010/02/18]
20. Bjelakovic G, Nikolova D, Bjelakovic M, et al. Vitamin D supplementation for chronic liver diseases in adults. *Cochrane Database Syst Rev* 2017;11:CD011564. doi: https://dx.doi.org/10.1002/14651858.CD011564.pub2
21. Shamseer L, Moher D, Clarke M, et al. Preferred reporting items for systematic review and meta-analysis protocols (PRISMA-P) 2015: elaboration and

For peer review only

explanation. 2015;349:g7647. doi: 10.1136/bmj.g7647 %J BMJ : British Medical Journal

22. The Scientific Advisory Committee on Nutrition. Vitamin D and Health: Public Health England 2016.

23. Jonathan J Deeks, Julian PT Higgins, Douglas G Altman. 9.5.2 Identifying and measuring heterogeneity. In: Douglas G Altman, ed. Cochrane Handbook for Systematic Reviews of Intervention Version 510 (updated March 2011): The Cochrane Collaboration 2011.

24. Jonathan AC Sterne, Matthias Egger, David Moher. 10.4.1 Funnel plots. In: Moher D, ed. Cochrane Handbook for Systematic Reviews of Intervention Version 510 (updated March 2011): The Cochrane Collaboration 2011.

25. Guyatt GH, Oxman AD, Vist GE, et al. GRADE: an emerging consensus on rating quality of evidence and strength of recommendations. *Bmj* 2008;336(7650):924-26. doi: 10.1136/bmj.39489.470347.AD

26. Pearce SH, Cheetham TD. Diagnosis and management of vitamin D deficiency. 2010;340:b5664. doi: 10.1136/bmj.b5664 %J BMJ

27. Institute of Medicine. Dietary Reference Intakes for Calcium and Vitamin D. Washington (DC): National Academies Press (US) 2011.

28. Holick MF, Binkley NC, Bischoff-Ferrari HA, et al. Evaluation, treatment, and prevention of vitamin D deficiency: an Endocrine Society clinical practice guideline. *The Journal of clinical endocrinology and metabolism* 2011;96(7):1911-30. doi: 10.1210/jc.2011-0385 [published Online First: 2011/06/08]

For peer review only

29. Hanley DA, Cranney A, Jones G, et al. Vitamin D in adult health and disease: a review and guideline statement from Osteoporosis Canada (summary). *Cmaj* 2010;182(12):1315-9. doi: 10.1503/cmaj.091062 [published Online First: 2010/07/14]

For peer review only

Supplementary Table 1: List of oral vitamin D analogues

Name	Indication	Brand name
Paricalcitol (19-nor-1α,25(OH)2D2)	Secondary hyperparathyroidism	Zemplar® (Abbott)
Doxercalciferol (1α(OH)D2)	Secondary hyperparathyroidism	Hectorol® (Genzyme corp)
Falecalcitriol (26,27 F6-1α,25(OH)2D3)	Secondary hyperparathyroidism (Japan only)	Fulstan® (Dainippon Sumitomo) and Hornel® (Taisho Yakuhin)
Maxacalcitol (22oxa-1α,25(OH)2D3)	Secondary hyperparathyroidism and psoriasis (Japan only)	Oxarol® (Chugai Pharmaceutical)
Eldecalcitol (2β-(3-hydroxypropoxy)-1α,25(OH)2D3)	Osteoporosis (Japan only)	Edirol® (Chugai Pharmaceutical)

For peer review only

Supplementary Table 2: Search strategies in Medline (Ovid)

Item	Search Terms
Herpesviruses	
1	(HHV adj1 ("1" or "2" or "3" or "4" or "5" or "6" or "7" or "8")) or (HHV?1 or HHV?2 or HHV?3 or HHV?4 or HHV?5 or HHV?6 or HHV?7 or HHV?8)
2	exp Herpesviridae Infections/ or exp Herpesviridae/
3	herpes* or herpesvir*
4	exp Herpes Simplex/
5	herpes simplex
6	HSV-1 or HSV?1
7	Cold sore*
8	exp Herpes Labialis/
9	Herpes Labialis
10	Herpes* adj2 Labial*
11	Hsv-2 or HSV?2 or (genit* herpes* or genit* sores)
12	exp Herpes Zoster/
13	herpes zoster
14	vz virus* or VZV
15	varicella?
16	chickenpox or Chicken?pox
17	shingle*
18	neuralgia adj1 post?herpetic)
19	Epstein-Barr virus* or (burkitt lymphoma adj2 virus*) or e?b virus* or ((eb or e-b) adj virus*) or ebv or Epstein?barr virus* or infectious mononucleosis or glandular fever
20	Cytomegalovirus* or CMV
21	salivary gland virus* or hblv or betaherpesvirus or b-lymphotropic virus* or cihhv or icihhv or Roseolovirus or Roseola Infantum or Exanthema Subitum
22	Kaposi* sarcoma-associated herpesvir* or KSHV or Primary Effusion Lymphoma
23	ac?clovir or Zovirax or valac?clovir or valtrex or famc?clovir or famvir or penc?clovir or ganc?clovir or cidofovir or foscarnet* or valganc?clovir or lubocavir or brivudin or Docosanol or Sorivudine or Idoxuridine or Trifluridine
24	1 or 2 or 3 or 4 or 5 or 6 or 7 or 8 or 9 or 10 or 11 or 12 or 13 or 14 or 15 or 16 or 17 or 18 or 19 or 20 or 21 or 22 or 23
Vitamin D	
25	exp Vitamin D/ or exp Vitamin D Deficiency/
26	(vitamin* or hydroxyvitamin* or dihydroxyvitamin*) adj1 d*
27	c?olecalciferol* or calciol* or calcitriol* or ergocalciferol* or calcifediol* or calcid?ol* or calciferol* or ercalcidiol* or ercalcitriol* or hydroxyergocalciferol* or hydroxycalciferol* or hydroxyc?olecalciferol* or dihydroxyc?olecalciferol* or ergosterol
28	(dihydrotachyst* or alphacalcid?ol or alfacalcid?ol or Falecalcitriol* or Maxacalcitol* or Oxacalcitriol* or Paricalcitol* or Lexacalcitol* or Seocalcitol* or Calcipotriol* or vitamin D analogue*
29	exp Familial Hypophosphatemic Rickets/ or exp Rickets, Hypophosphatemic/ or exp Rickets/
30	Rickets
31	rachitides
32	rachitis
33	25 or 26 or 27 or 28 or 29 or 30 or 31 or 32
34	24 and 33

For peer review only

Supplementary Table 3: modified keywords for grey literature search

Search Concept	Keywords Groups
Herpesviruses	Group 1: herpesvir* OR herpes* OR HHV* OR HSV* OR Cold sore* OR varicella* OR shingle*<br>Group 2: chickenpox OR post-herpetic neuralgia OR Burkitt lymphoma OR EBV OR Epstein-Barr<br>Group 3: Cytomegalovirus* or CMV OR Kaposi* sarcoma-associated herpesvir* or KSHV OR famc*
Vitamin D	Group 4: "vitamin D" or hydroxyvitamin* or dihydroxyvitamin*<br>Group 5: calcitriol* OR cholecalciferol OR ergocalciferol* OR Calcidol<br>Group 6: calcifer* OR ergosterol OR rickets OR Calcipotriol* OR vitamin D analogue
Combination of concepts	(Group 1, Group 2, Group 3) x (Group 4, Group 5, Group 6)

For peer review only

Supplementary Table 4: Domains of risk of bias assessment

Domains	Explanation
Confounders	Not adjusted for confounders
Selection bias	Intervention studies: random sequence generation and allocation concealment<br>Observational studies: and participation bias, selection of participants, and selection of controls
Misclassification of exposure and outcomes	Differential: recall bias, observer bias<br>Non-differential: ascertainment bias
Performance bias and detection bias	Blinding of participants, personnel and outcome assessment
Attrition bias	Incomplete outcome data
Bias due to Missing Data	Differential loss to follow up and exclusion of individuals with missing data
Reporting bias	Selective reporting
Reverse causation	Defined outcomes prior to the exposures

For peer review only

Reporting checklist for protocol of a systematic review.

Based on the PRISMA-P guidelines.

Instructions to authors

Complete this checklist by entering the page numbers from your manuscript where readers will find each of the items listed below.

Your article may not currently address all the items on the checklist. Please modify your text to include the missing information. If you are certain that an item does not apply, please write "n/a" and provide a short explanation.

Upload your completed checklist as an extra file when you submit to a journal.

In your methods section, say that you used the PRISMA-Preporting guidelines, and cite them as:

Moher D, Shamseer L, Clarke M, Ghersi D, Liberati A, Petticrew M, Shekelle P, Stewart LA. Preferred Reporting Items for Systematic Review and Meta-Analysis Protocols (PRISMA-P) 2015 statement. Syst Rev. 2015;4(1):1.

		Reporting Item	Page Number
**Title**			
Identification	#1a	Identify the report as a protocol of a systematic review	1
Update	#1b	If the protocol is for an update of a previous systematic review, identify as such	n/a

For peer review only

**Registration**			
	#2	If registered, provide the name of the registry (such as PROSPERO) and registration number	4
**Authors**			
Contact	#3a	Provide name, institutional affiliation, e-mail address of all protocol authors; provide physical mailing address of corresponding author	1
Contribution	#3b	Describe contributions of protocol authors and identify the guarantor of the review	15
**Amendments**			
	#4	If the protocol represents an amendment of a previously completed or published protocol, identify as such and list changes; otherwise, state plan for documenting important protocol amendments	n/a
**Support**			
Sources	#5a	Indicate sources of financial or other support for the review	18
Sponsor	#5b	Provide name for the review funder and / or sponsor	18
Role of sponsor or funder	#5c	Describe roles of funder(s), sponsor(s), and / or institution(s), if any, in developing the protocol	n/a
**Introduction**			
Rationale	#6	Describe the rationale for the review in the context of what is	6

For peer review only

		already known	
Objectives	#7	Provide an explicit statement of the question(s) the review will address with reference to participants, interventions, comparators, and outcomes (PICO)	7
**Methods**			
Eligibility criteria	#8	Specify the study characteristics (such as PICO, study design, setting, time frame) and report characteristics (such as years considered, language, publication status) to be used as criteria for eligibility for the review	8
Information sources	#9	Describe all intended information sources (such as electronic databases, contact with study authors, trial registers or other grey literature sources) with planned dates of coverage	10
Search strategy	#10	Present draft of search strategy to be used for at least one electronic database, including planned limits, such that it could be repeated	10
Study records - data management	#11a	Describe the mechanism(s) that will be used to manage records and data throughout the review	10
Study records - selection process	#11b	State the process that will be used for selecting studies (such as two independent reviewers) through each phase of the review (that is, screening, eligibility and inclusion in meta-analysis)	11
Study records - data collection	#11c	Describe planned method of extracting data from reports (such as piloting forms, done independently, in duplicate), any	11

For peer review only

process		processes for obtaining and confirming data from investigators	
Data items	#12	List and define all variables for which data will be sought (such as PICO items, funding sources), any pre-planned data assumptions and simplifications	11
Outcomes and prioritization	#13	List and define all outcomes for which data will be sought, including prioritization of main and additional outcomes, with rationale	12
Risk of bias in individual studies	#14	Describe anticipated methods for assessing risk of bias of individual studies, including whether this will be done at the outcome or study level, or both; state how this information will be used in data synthesis	12
Data synthesis	#15a	Describe criteria under which study data will be quantitatively synthesised	13
Data synthesis	#15b	If data are appropriate for quantitative synthesis, describe planned summary measures, methods of handling data and methods of combining data from studies, including any planned exploration of consistency (such as I2, Kendall's τ)	13
Data synthesis	#15c	Describe any proposed additional analyses (such as sensitivity or subgroup analyses, meta-regression)	13
Data synthesis	#15d	If quantitative synthesis is not appropriate, describe the type of summary planned	13
Meta-bias(es)	#16	Specify any planned assessment of meta-bias(es) (such as publication bias across studies, selective reporting within	13

For peer review only

		studies)	
Confidence in cumulative evidence	#17	Describe how the strength of the body of evidence will be assessed (such as GRADE)	13

The PRISMA-P checklist is distributed under the terms of the Creative Commons Attribution License CC-BY 4.0. This checklist was completed on 22. May 2019 using https://www.goodreports.org/, a tool made by the EQUATOR Network in collaboration with Penelope.ai

For peer review only

Vitamin D deficiency or supplementation and the risk of human herpesvirus infections or reactivation: a systematic review protocol

Journal:	*BMJ Open*
Manuscript ID	bmjopen-2019-031867.R2
Article Type:	Protocol
Date Submitted by the Author:	05-Sep-2019
Complete List of Authors:	Lin, Liang-Yu; London School of Hygiene and Tropical Medicine Faculty of Epidemiology and Population Health, Department of Non-communicable Disease Epidemiology<br>Bhate, Ketaki; London School of Hygiene and Tropical Medicine Faculty of Epidemiology and Population Health, Department of Non-communicable Disease Epidemiology<br>Forbes, Harriet; London School of Hygiene and Tropical Medicine Faculty of Epidemiology and Population Health, Department of Non-communicable Disease Epidemiology<br>Smeeth, Liam; London School of Hygiene and Tropical Medicine Faculty of Epidemiology and Population Health, Department of Non-communicable Disease Epidemiology<br>Warren-Gash, Charlotte; London School of Hygiene and Tropical Medicine Faculty of Epidemiology and Population Health, Department of Non-communicable Disease Epidemiology<br>Langan, Sinead; London School of Hygiene and Tropical Medicine Faculty of Epidemiology and Population Health, Department of Non-communicable Disease Epidemiology
**Primary Subject Heading**:	Infectious diseases
Secondary Subject Heading:	Nutrition and metabolism
Keywords:	systematic review, herpesviridae, vitamin D deficiency, vitamin D supplementation

Title: Vitamin D deficiency or supplementation and the risk of human herpesvirus infections or reactivation: a systematic review protocol

Authors Contact

Dr Liang-Yu Lin[1]. LSHTM liang-yu.lin@lshtm.ac.uk ORCID: https://orcid.org/0000-0003-4720-6738

Dr Ketaki Bhate[1]. LSHTM Ketaki.Bhate@lshtm.ac.uk

ORCID: https://orcid.org/0000-0001-5509-4428

Dr Harriet Forbes[1]. LSHTM Harriet.Forbes@lshtm.ac.uk

ORCID: https://orcid.org/0000-0001-6888-2212

Professor Liam Smeeth[1]. LSHTM Liam.Smeeth@lshtm.ac.uk

ORCID: https://orcid.org/0000-0002-9168-6022

Dr Charlotte Warren-Gash[1]*. LSHTM Charlotte.Warren-Gash1@lshtm.ac.uk

ORCID: https://orcid.org/0000-0003-4524-3180

Professor Sinéad M Langan[1]*. LSHTM Sinead.Langan@LSHTM.ac.uk

ORCID: https://orcid.org/0000-0002-7022-7441

* The senior authors contributed equally to this work

Authors' Affiliations:

1. Faculty of Epidemiology & Population Health, London School of Hygiene and Tropical Medicine, London, UK

For peer review only

Correspondence to: Professor Sinéad M Langan

LSHTM, Keppel Street, London, WC1E 7HT, United Kingdom

Tel: 0207 9272680, Sinead.Langan@LSHTM.ac.uk

Word count: 2672

Keywords: systematic review, herpesviridae, vitamin D deficiency, vitamin D supplementation

For peer review only

ABSTRACT:

Introduction:

Human herpesviruses induce lifelong latent infections and may reactivate as the immune system deteriorates. Recent studies have suggested that vitamin D, an essential element of bone health, may have some effect of protecting against infections, but investigations of its potential to prevent herpesvirus infection or reactivation are limited. We will review the current literature examining vitamin D and the risk of herpesvirus infections or reactivation.

Methods and analysis:

Our systematic review will address two research questions: 1. Do deficient/insufficient serum vitamin D levels increase the risk of herpesvirus infections, and 2. Does vitamin D supplementation protect against herpesvirus infections? We will include only intervention studies with control groups, cohort studies and case-control studies. We will use subject headings and keywords to search for synonyms of “vitamin D” and “herpesviruses” (including herpes simplex virus type 1 and 2, varicella-zoster virus, cytomegalovirus, Epstein-Barr virus and human herpesviruses type 6,7 and 8) in Medline, Embase, Global Health, Web of Science, Scopus, and Cochrane Central Register of Controlled Trials, and the grey literature databases Prevention Information & Evidence eLibrary, Open Grey, EThOS, and BASE from inception to 31 August 2019. References to the included articles and relevant systematic reviews will also be examined. Two reviewers will independently screen the study titles and abstracts, and examine the full texts to decide the final eligibility. They will independently extract data

For peer review only

from the studies and assess bias using the Cochrane Collaboration approach. A third researcher will solve any discrepancies. The results will be narratively synthesized; if an adequate number of studies is included and the homogeneity between studies is acceptable, a meta-analysis will be performed. We will assess the quality of evidence using the Grading of Recommendations, Assessment, Development and Evaluation (GRADE) framework, and display the results in a summary of findings table.

Ethics and dissemination

Ethical review is not required for a systematic review. We will publish the results in a peer-review journal. Any amendments to the protocol will be recorded in the supplementary section.

PROSPERO registration number:

CRD42019130153

For peer review only

STRENGTHS AND LIMITATION OF THIS STUDY:

- This systematic review will be undertaken following the predefined population, exposure, comparator and outcome framework.
- All available databases, including six major databases and four grey literature databases, will be searched to obtain all eligible studies.
- The summarized results will improve our understanding of the current evidence of the possible association between vitamin D and herpesvirus infection/reactivation.
- The number of sufficient eligible studies may be inadequate, especially for some viruses that are harder to diagnose; also, the included studies may not have an adequate quality of evidence to answer the research questions.

For peer review only

INTRODUCTION

Rationale

Herpesviruses are a group of double-stranded DNA viruses that infect humans and some animals. After infecting their hosts, these viruses cannot be eradicated; instead, they establish latency and persist for life. As the host's immunity declines, these viruses can reactivate to induce various symptoms. There are nine human herpesviruses (Table 1).[1] Reactivation of herpesviruses may induce serious complications. For instance, herpes simplex viruses 1 (HSV-1) can lead to herpetic keratitis, which is the major cause of blindness in developed countries;[2] Epstein-Barr virus (EBV) may induce nasopharyngeal cancer;[3] and Varicella zoster virus (VZV) would cause herpes zoster and post-herpetic neuralgia, which increases financial burdens especially for people older than aged 65 years.[4] Consequently, investigating immunomodulatory factors associated with infection or reactivation of this virus family is important.

Vitamin D is mainly endogenously synthesized by the skin after sun exposure and can be supplied through dietary intake and supplementation. It plays an important role in absorbing calcium and phosphate, which are essential for bone health.[5] Recently, some studies have indicated that vitamin D may have potential immunomodulatory effects associated with the regulation of antimicrobial peptides (AMPs).[6] In previous cell studies, vitamin D induced gene expression of an AMP named Cathelicidin. In response to pathogen exposure, immune cells such as monocytes or macrophages, upregulate vitamin D receptors and enzymes to increase the production of Cathelicidin.[7-9] In addition, evidence suggests that vitamin D has some effects on the adaptive immune

For peer review only

system. Vitamin D suppresses CD4+ Th 1 lymphocytes and increases Th 2 lymphocytes, and it also intensifies the effect of regulatory T lymphocyte responses.[10 11] Regarding the effects of vitamin D associated AMPs on herpesviruses, a cell study indicated that Cathelicidin decreased HSV-1 viral titres isolated from keratoconjunctivitis patients;[12] furthermore, another cell study also showed that vitamin D supplementation reduced HSV-1 viral load and mRNA expression in HSV-1 infected cells.[13]

Vitamin D also shows some anti-infective potential in epidemiological studies. A meta-analysis using original patient data from 25 randomized controlled trials showed that among the general population, vitamin D supplementation reduced the risk of acute respiratory infections.[14] Furthermore, there is some evidence to suggest an anti-infective effect of vitamin D in specific patient groups, such as patients with chronic kidney disease (CKD), HIV or hepatitis C virus (HCV) infection. Among CKD patients receiving dialysis, a case-control study indicated that the risk of herpes zoster reactivation was significantly lower in those who received vitamin D supplementation;[15] another meta-analysis also showed that CKD patients with higher or normal serum vitamin D levels had a lower risk of infection.[16] Among HIV infected patients, lower serum vitamin D levels were also associated with a higher risk of clinical progression to AIDS and all-cause mortality in a cohort study,[17] while vitamin D supplementation didn’t affect mortality, CD4 cell count or viral load.[18] For HCV infected patients, serum vitamin D levels were inversely associated with the grade of liver inflammation and the stage of fibrosis,[19] while no protective effect of vitamin D supplementation was seen in a meta-analysis of clinical trials.[20] However, the effect of vitamin D on herpesvirus infection or reactivation in the general population is unclear.

For peer review only

In this study, we will comprehensively review studies of the effect of serum vitamin D levels or the use of oral vitamin D supplementation on infection with or reactivation of any of the eight human herpesviruses.

OBJECTIVE

This review aims to explore the association between vitamin D and herpesviruses. The proposed systematic review will address two primary research questions:

1. Is serum vitamin D deficiency/insufficiency associated with an increased risk of infection with or reactivation of human herpesviruses?
2. Does oral vitamin D supplementation protect against infection with or reactivation of human herpesviruses?

For peer review only

METHODS

This study protocol will be reported according to the Preferred Reporting Items for Systematic Review and Meta-Analysis Protocols (PRISMA-P).[21]

Patient and Public Involvement

Patients and or the public were not involved in this systematic review protocol.

Eligibility Criteria

Study Design and Characteristics

To identify the possible causal association between vitamin D and herpesvirus infections, we will review observational studies, including cohort and case-control studies, and intervention studies with any type of control group, either placebo, active comparator or no treatment, including randomized or non-randomized controlled trials. Descriptive studies, ecological studies, cross-sectional studies, case reports and case series will not be included. If a systematic review relevant to our topic is found, we will review its references.

Participants

Only human studies will be included in our review, and animal or cell studies will be excluded. Studies from all age groups or involving patients with any immune status are eligible.

For peer review only

Exposure

We have two exposures of interest for each research question in our review. For the first research question, the exposures are deficient or insufficient serum vitamin D levels in the participants. We will include articles in which the serum vitamin D levels are identified as deficient or insufficient. Notably, no consensus exists about serum vitamin D levels, and different studies may use different definitions (Table 2). If eligible studies provide original values for the serum 25(OH)D levels, we will define vitamin D deficiency as 25(OH)D < 25 nmol/L and insufficiency as 25(OH)D in the range from 25-50 nmol/L in accordance with the standards of Public Health England[22].

For the second research question, the intervention is oral vitamin D supplementation or oral vitamin D analogue treatment used for secondary hyperparathyroidism or osteoporosis (Supplementary Table 1). We will exclude studies using topical vitamin D analogues, because their effects on systemic serum vitamin D levels are unknown.

Comparators

The comparator groups for vitamin D insufficiency and deficiency are those with sufficient serum vitamin D levels. For those receiving oral vitamin D supplementation or treatment, the comparators are those without vitamin D supplementation or treatment or receiving placebo or another active comparator, respectively.

Outcomes

The outcomes will be infection with or reactivation of all eight human herpesviruses listed in Table 1. Infection with and reactivation of herpesviruses are defined using

For peer review only - http://bmjopen.bmj.com/site/about/guidelines.xhtml

clinical or laboratory criteria. The clinical criteria include patients presenting with classical symptoms, for instance, such as the painful rash of herpes zoster, or a diagnosis recorded by physicians. Laboratory criteria include using laboratory techniques to confirm the diagnosis, such as evaluation of the serum viral load by PCR. Studies reporting only serum antibodies against herpesviruses will not be included.

Information sources

We will search the following database from inception to 31 August 2019: Medline (Ovid), EMBASE (Ovid), Web of Science, Scopus, Cochrane Library, and Global Health (Ovid). To enhance the sensitivity of our search and reduce the risk of publication bias, we will also search grey literature databases such as Open Grey, Prevention Information & Evidence eLibrary, BASE, EThOS and the clinical trials register at ClinicalTrials.gov.

Search strategy

We will search for synonyms of “human herpesviruses” and “vitamin D” using both controlled vocabularies and keywords in each database. A search strategy in Medline (Ovid) is listed in Supplementary Table 2. The subject headings will be modified for different databases. The results will be combined using the Boolean logic operator “AND.” For some database with limited search functions, such as single line search, keywords will be split into small sections to fulfil the requirement (Supplementary Table 3). In addition to searching electronic databases, we will manually search for the references lists of the included articles and relevant systematic reviews.

For peer review only

Study records

Data management

One researcher will import the search results from different databases into the citation management software EndNote X9 (Clarivate Analytics, version 9.1/2019). Duplicated results will be identified and deleted.

Selection process

Two researchers will independently screen the titles and abstracts of all identified studies. We will obtain full texts of all studies fulfilling the review criteria, and the two researchers will screen all articles to establish their eligibility for inclusion. Any discrepancy in the reviewing process will be adjudicated by the third researcher.

Data collection process

Data extraction for the first three studies will be performed by two independent researchers to ensure the integrity of the process; then, one researcher will extract data from the remaining studies. If any data are missing or unclear, we will contact the authors for further clarification and information.

Data items

We will summarize and extract data from the included studies using a Population, Exposure/Intervention, Comparator, Outcomes, and Study characteristics framework as follows:

For peer review only

179 1. Population: sample sizes of each study, inclusion or exclusion criteria for the participants, and demographic characteristics, such as sex, age, or immune status
182 2. Exposure 1: insufficient or deficit serum vitamin D levels of the study participants Exposure 2: oral vitamin D supplementation or vitamin D analogue
184 3. Comparator 1: sufficient serum vitamin D levels among the participants Comparator 2: without vitamin D supplementation or vitamin D analogue use
186 4. Outcomes: definition of herpesvirus infection/reactivation, i.e. clinically diagnosed or laboratory-confirmed herpesvirus infections; the number of study subjects with the outcomes
189 5. Study characteristics: publication details (authors, publication year, country, and journal), study designs, confounders measured, confounders adjusted and study results

192 **Outcomes and prioritization**

193 The outcomes of the studies are herpesvirus infections or reactivation. The disease can be diagnosed either clinically or through laboratory confirmation. Some herpesvirus infections, such as herpes zoster, may lead to characteristic clinical symptoms, and some laboratory approaches, such as PCR, can detect the viral load, which can also be proof of herpesvirus infection/reactivation. Studies measuring serum anti-herpesvirus antibodies will not be included, because IgG antibodies detected in the absence of clinical symptoms cannot ascertain the timing of infection. Regarding the study results, our focus is on the incidence of herpesviruses infection or reactivation. We will report the outcome definition used for each study, and will extract data on the appropriate

For peer review only

effect measure. The effect measures will include odds ratios for case-control studies and risk, rate or hazard ratios for cohort studies or clinical trials. If studies report only continuous outcomes such as viral load, then we will summarise these using means or medians as appropriate.

Risk of bias in individual studies

We will assess the risk of bias from the included studies using a template form based on the Cochrane approach for trials and observational studies, and all relevant domains of bias will be assessed for different study types. For randomized controlled trials, we will evaluate bias due to the following sources: 1. random sequence generation; 2. allocation concealment; 3. blinding of participants, personnel, or assessment; 4. incomplete outcome data; and 5. selective reporting. For observational studies, we will consider bias due to the following sources: 1. confounding factors; 2. selection of participants or controls; 3. differential and non-differential misclassification of the exposure or outcome; 4. reverse causation; and 5. missing data (Supplementary Table 4). A piloted form will be tested by applying it to extract data from the first three studies. To ensure the quality and consistency of the risk of bias assessment, the first three studies will be evaluated by two independent researchers. Then, one researcher will complete the evaluation of the remaining included studies. Any discrepancies will be examined by the third researcher.

Data synthesis and meta-bias (es)

We will comprehensively search different databases including grey literature databases to minimize bias in our search. We will use a narrative synthesis to summarize the data

For peer review only

and results from the eligible studies included in our review. Since each herpesvirus subtypes have different pathogenic pathways, we will display the results separately by virus. If an adequate number of studies with acceptable homogeneity are included, a meta-analysis will be performed to integrate the study results. We will decide whether to use fixed- or random-effects models by considering the I^2 value for heterogeneity; values >50% will be considered to represent substantial heterogeneity[23]. If the number of included studies is sufficient, we will carry out subgroup analyses of subjects with baseline vitamin D status, intervention trials, study durations, different ages, immune statuses, or latitude to explore sources of heterogeneity. A funnel plot will be used to present the distribution of studies and examine any possible publication bias[24]. All statistical analyses will be performed by using STATA version 15.

Confidence in cumulative evidence

We will evaluate the quality of evidence using the Grading of Recommendations, Assessment, Development and Evaluations (GRADE) framework[25]. We will evaluate the design and risk of bias across all included studies. Studies with significant effects, strong dose responses, or that are well adjusted for plausible confounders will receive higher grades, whereas those with a higher risk of bias, inconsistency, indirectness, or imprecision will be downgraded. We will conclude the quality of evidence using a 'Summary of Findings' table and assign each outcome a quality rank of "high", "moderate", "low" or "very low" for the level of confidence.

For peer review only

Ethics and dissemination

Ethical review is not required for a systematic review. We will publish the results in a peer-review journal. Any changes in the study protocol will be recorded and reported. Any revision of the protocol will be recorded in the supplementation of the final published review.

For peer review only

AUTHORS' CONTRIBUTION

LYL contributed to the design of the study, drafted the introduction, methods and analysis and revised the protocol according to other authors' comments; CWG contributed to the design of the study, made critical comments on the protocol and revised the paper critically; SML contributed to the design of the study, made critical comments on the protocol and revised the paper critically; KB contributed to the design of the study and revised the paper critically; HJF contributed to the design of the study and revised the paper critically; LS contributed to the design of the study and revised the paper critically. All authors approved the final version of the protocol.

For peer review only

Table 1: List of human herpesviruses

Common Name	Abbreviation
Herpes simplex virus type 1 (HSV-1)	HHV-1
Herpes simplex virus type 2 (HSV-2)	HHV-2
Varicella-zoster virus (VZV)	HHV-3
Epstein-Barr virus (EBV)	HHV-4
Cytomegalovirus (CMV)	HHV-5
HHV-6 variant A	HHV-6A
HHV-6 variant B	HHV-6B
HHV-7	HHV-7
Kaposi's sarcoma–associated HV (KSHV)	HHV-8

For peer review only

Table 2: The normal range of serum vitamin D levels adapted in different studies

Study	Deficiency (nmol/L)	Insufficiency (nmol/L)	Adequate (nmol/L)
Pearce and Cheetham, 2010[26]	<25	25-50	50-75
Institute of Medicine, 2011[27]	<30	30-50	≥50
Holick et al., 2011[28]	<50	52.5-72.5	
Hanley et al., 2010[29]	<25	25-75	

* 1 nmol/L = 0.4 ng/mL

For peer review only

SUPPORT

This research received no specific grant from any funding agency in the public, commercial or not-for-profit sectors.

FUNDING STATEMENT

LYL is funded by the scholarship of government sponsorship for overseas study by the Ministry of Education Republic of China (Taiwan). CWG is supported by a Wellcome Intermediate Clinical Fellowship (201440_Z_16_Z). SML is funded by a Wellcome Senior Clinical Fellowship in Science (205039/Z/16/Z). KB is supported by a National Institute of Health Research (NIHR) Doctoral Research Fellowship (DRF-2018-11-ST2-066).

COMPETING INTERESTS STATEMENT:

The authors declare that they have no known conflicts of interest.

For peer review only

References:

1. Knipe DM, Howley P. Fields Virology: Wolters Kluwer Health 2013:1806.

2. Liesegang TJ. Herpes simplex virus epidemiology and ocular importance. *Cornea* 2001;20(1):1-13. [published Online First: 2001/02/24]

3. Young LS, Yap LF, Murray PG. Epstein-Barr virus: more than 50 years old and still providing surprises. *Nat Rev Cancer* 2016;16(12):789-802. doi: 10.1038/nrc.2016.92

4. Scott FT, Johnson RW, Leedham-Green M, et al. The burden of Herpes Zoster: a prospective population based study. *Vaccine* 2006;24(9):1308-14. doi: 10.1016/j.vaccine.2005.09.026 [published Online First: 2005/12/15]

5. Holick MF. Vitamin D deficiency. *The New England journal of medicine* 2007;357(3):266-81. doi: 10.1056/NEJMra070553 [published Online First: 2007/07/20]

6. Zasloff M. Antimicrobial peptides of multicellular organisms. *Nature* 2002;415(6870):389-95. doi: 10.1038/415389a [published Online First: 2002/01/25]

7. Gombart AF, Borregaard N, Koeffler HP. Human cathelicidin antimicrobial peptide (CAMP) gene is a direct target of the vitamin D receptor and is strongly up-regulated in myeloid cells by 1,25-dihydroxyvitamin D3. *Faseb J* 2005;19(9):1067-77. doi: 10.1096/fj.04-3284com [published Online First: 2005/06/30]

8. Wang TT, Nestel FP, Bourdeau V, et al. Cutting edge: 1,25-dihydroxyvitamin D3 is a direct inducer of antimicrobial peptide gene expression. *J Immunol*

For peer review only

2004;173(5):2909-12. doi: 10.4049/jimmunol.173.5.2909 [published Online First: 2004/08/24]

9. Weber G, Heilborn JD, Chamorro Jimenez CI, et al. Vitamin D induces the antimicrobial protein hCAP18 in human skin. *The Journal of investigative dermatology* 2005;124(5):1080-2. doi: 10.1111/j.0022-202X.2005.23687.x [published Online First: 2005/04/28]

10. Cantorna MT, Yu S, Bruce D. The paradoxical effects of vitamin D on type 1 mediated immunity. *Molecular aspects of medicine* 2008;29(6):369-75. doi: 10.1016/j.mam.2008.04.004 [published Online First: 2008/05/04]

11. Mann EH, Pfeffer PE, Hawrylowicz CM. Vitamin D and Adaptive Immunology in Health and Disease. Vitamin D. Fourth Edi ed: Elsevier, 2018:937-49.

12. Gordon YJ, Huang LC, Romanowski EG, et al. Human cathelicidin (LL-37), a multifunctional peptide, is expressed by ocular surface epithelia and has potent antibacterial and antiviral activity. *Current eye research* 2005;30(5):385-94. doi: 10.1080/02713680590934111 [published Online First: 2005/07/16]

13. Kumar A, Singh MP, Kumar RS, et al. 25-Hydroxyvitamin D3 and 1,25 Dihydroxyvitamin D3 as an Antiviral and Immunomodulator Against Herpes Simplex Virus-1 Infection in HeLa Cells. *Viral Immunol* 2018;31(8):589-93. doi: 10.1089/vim.2018.0026 [published Online First: 2018/05/25]

14. Martineau AR, Jolliffe DA, Hooper RL, et al. Vitamin D supplementation to prevent acute respiratory tract infections: systematic review and meta-analysis of individual participant data. *Bmj* 2017;356:i6583. doi: https://dx.doi.org/10.1136/bmj.i6583

For peer review only

15. Chao CT, Lai CF, Huang JW. Risk factors for herpes zoster reactivation in maintenance hemodialysis patients. *Eur* 2012;23(8):711-5. doi: https://dx.doi.org/10.1016/j.ejim.2012.08.005

16. Su G, Liu Z, Qin X, et al. Vitamin D deficiency and treatment versus risk of infection in end-stage renal disease patients under dialysis: a systematic review and meta-analysis. *Nephrol Dial Transplant* 2018 doi: 10.1093/ndt/gfy216 [published Online First: 2018/07/31]

17. Viard J-Pa, Souberbielle J-Cb, Kirk Oc, et al. Vitamin D and clinical disease progression in HIV infection: results from the EuroSIDA study. *Aids* 2011;25(10):1305-15.

18. Visser ME, Durao S, Sinclair D, et al. Micronutrient supplementation in adults with HIV infection. *Cochrane Database Syst Rev* 2017;5:CD003650. doi: https://dx.doi.org/10.1002/14651858.CD003650.pub4

19. Petta S, Camma C, Scazzone C, et al. Low vitamin D serum level is related to severe fibrosis and low responsiveness to interferon-based therapy in genotype 1 chronic hepatitis C. *Hepatology* 2010;51(4):1158-67. doi: 10.1002/hep.23489 [published Online First: 2010/02/18]

20. Bjelakovic G, Nikolova D, Bjelakovic M, et al. Vitamin D supplementation for chronic liver diseases in adults. *Cochrane Database Syst Rev* 2017;11:CD011564. doi: https://dx.doi.org/10.1002/14651858.CD011564.pub2

21. Shamseer L, Moher D, Clarke M, et al. Preferred reporting items for systematic review and meta-analysis protocols (PRISMA-P) 2015: elaboration and

For peer review only

explanation. 2015;349:g7647. doi: 10.1136/bmj.g7647 %J BMJ : British Medical Journal

22. The Scientific Advisory Committee on Nutrition. Vitamin D and Health: Public Health England 2016.

23. Jonathan J Deeks, Julian PT Higgins, Douglas G Altman. 9.5.2 Identifying and measuring heterogeneity. In: Douglas G Altman, ed. Cochrane Handbook for Systematic Reviews of Intervention Version 510 (updated March 2011): The Cochrane Collaboration 2011.

24. Jonathan AC Sterne, Matthias Egger, David Moher. 10.4.1 Funnel plots. In: Moher D, ed. Cochrane Handbook for Systematic Reviews of Intervention Version 510 (updated March 2011): The Cochrane Collaboration 2011.

25. Guyatt GH, Oxman AD, Vist GE, et al. GRADE: an emerging consensus on rating quality of evidence and strength of recommendations. *Bmj* 2008;336(7650):924-26. doi: 10.1136/bmj.39489.470347.AD

26. Pearce SH, Cheetham TD. Diagnosis and management of vitamin D deficiency. 2010;340:b5664. doi: 10.1136/bmj.b5664 %J BMJ

27. Institute of Medicine. Dietary Reference Intakes for Calcium and Vitamin D. Washington (DC): National Academies Press (US) 2011.

28. Holick MF, Binkley NC, Bischoff-Ferrari HA, et al. Evaluation, treatment, and prevention of vitamin D deficiency: an Endocrine Society clinical practice guideline. *The Journal of clinical endocrinology and metabolism* 2011;96(7):1911-30. doi: 10.1210/jc.2011-0385 [published Online First: 2011/06/08]

For peer review only

29. Hanley DA, Cranney A, Jones G, et al. Vitamin D in adult health and disease: a review and guideline statement from Osteoporosis Canada (summary). *Cmaj* 2010;182(12):1315-9. doi: 10.1503/cmaj.091062 [published Online First: 2010/07/14]

For peer review only

Supplementary Table 1: List of oral vitamin D analogues

Name	Indication	Brand name
Paricalcitol (19-nor-1α,25(OH)2D2)	Secondary hyperparathyroidism	Zemplar® (Abbott)
Doxercalciferol (1α(OH)D2)	Secondary hyperparathyroidism	Hectorol® (Genzyme corp)
Falecalcitriol (26,27 F6-1α,25(OH)2D3)	Secondary hyperparathyroidism (Japan only)	Fulstan® (Dainippon Sumitomo) and Hornel® (Taisho Yakuhin)
Maxacalcitol (22oxa-1α,25(OH)2D3)	Secondary hyperparathyroidism and psoriasis (Japan only)	Oxarol® (Chugai Pharmaceutical)
Eldecalcitol (2β-(3-hydroxypropoxy)-1α,25(OH)2D3)	Osteoporosis (Japan only)	Edirol® (Chugai Pharmaceutical)

For peer review only

Supplementary Table 2: Search strategies in Medline (Ovid)

Item	Search Terms
Herpesviruses	
1	(HHV adj1 ("1" or "2" or "3" or "4" or "5" or "6" or "7" or "8")) or (HHV?1 or HHV?2 or HHV?3 or HHV?4 or HHV?5 or HHV?6 or HHV?7 or HHV?8)
2	exp Herpesviridae Infections/ or exp Herpesviridae/
3	herpes* or herpesvir*
4	exp Herpes Simplex/
5	herpes simplex
6	HSV-1 or HSV?1
7	Cold sore*
8	exp Herpes Labialis/
9	Herpes Labialis
10	Herpes* adj2 Labial*
11	Hsv-2 or HSV?2 or (genit* herpes* or genit* sores)
12	exp Herpes Zoster/
13	herpes zoster
14	vz virus* or VZV
15	varicella?
16	chickenpox or Chicken?pox
17	shingle*
18	neuralgia adj1 post?herpetic)
19	Epstein-Barr virus* or (burkitt lymphoma adj2 virus*) or e?b virus* or ((eb or e-b) adj virus*) or ebv or Epstein?barr virus* or infectious mononucleosis or glandular fever
20	Cytomegalovirus* or CMV
21	salivary gland virus* or hblv or betaherpesvirus or b-lymphotropic virus* or cihhv or icihhv or Roseolovirus or Roseola Infantum or Exanthema Subitum
22	Kaposi* sarcoma-associated herpesvir* or KSHV or Primary Effusion Lymphoma
23	ac?clovir or Zovirax or valac?clovir or valtrex or famc?clovir or famvir or penc?clovir or ganc?clovir or cidofovir or foscarnet* or valganc?clovir or lubocavir or brivudin or Docosanol or Sorivudine or Idoxuridine or Trifluridine
24	1 or 2 or 3 or 4 or 5 or 6 or 7 or 8 or 9 or 10 or 11 or 12 or 13 or 14 or 15 or 16 or 17 or 18 or 19 or 20 or 21 or 22 or 23
Vitamin D	
25	exp Vitamin D/ or exp Vitamin D Deficiency/
26	(vitamin* or hydroxyvitamin* or dihydroxyvitamin*) adj1 d*
27	c?olecalciferol* or calciol* or calcitriol* or ergocalciferol* or calcifediol* or calcid?ol* or calciferol* or ercalcidiol* or ercalcitriol* or hydroxyergocalciferol* or hydroxycalciferol* or hydroxyc?olecalciferol* or dihydroxyc?olecalciferol* or ergosterol
28	(dihydrotachyst* or alphacalcid?ol or alfacalcid?ol or Falecalcitriol* or Maxacalcitol* or Oxacalcitriol* or Paricalcitol* or Lexacalcitol* or Seocalcitol* or Calcipotriol* or vitamin D analogue*
29	exp Familial Hypophosphatemic Rickets/ or exp Rickets, Hypophosphatemic/ or exp Rickets/
30	Rickets
31	rachitides
32	rachitis
33	25 or 26 or 27 or 28 or 29 or 30 or 31 or 32
34	24 and 33

For peer review only

Supplementary Table 3: modified keywords for grey literature search

Search Concept	Keywords Groups
Herpesviruses	Group 1: herpesvir* OR herpes* OR HHV* OR HSV* OR Cold sore* OR varicella* OR shingle*<br>Group 2: chickenpox OR post-herpetic neuralgia OR Burkitt lymphoma OR EBV OR Epstein-Barr<br>Group 3: Cytomegalovirus* or CMV OR Kaposi* sarcoma-associated herpesvir* or KSHV OR famc*
Vitamin D	Group 4: "vitamin D" or hydroxyvitamin* or dihydroxyvitamin*<br>Group 5: calcitriol* OR cholecalciferol OR ergocalciferol* OR Calcidol<br>Group 6: calcifer* OR ergosterol OR rickets OR Calcipotriol* OR vitamin D analogue
Combination of concepts	(Group 1, Group 2, Group 3) x (Group 4, Group 5, Group 6)

For peer review only

Supplementary Table 4: Domains of risk of bias assessment

Domains	Explanation
Confounders	Not adjusted for confounders
Selection bias	Intervention studies: random sequence generation and allocation concealment<br>Observational studies: and participation bias, selection of participants, and selection of controls
Misclassification of exposure and outcomes	Differential: recall bias, observer bias<br>Non-differential: ascertainment bias
Performance bias and detection bias	Blinding of participants, personnel and outcome assessment
Attrition bias	Incomplete outcome data
Bias due to Missing Data	Differential loss to follow up and exclusion of individuals with missing data
Reporting bias	Selective reporting
Reverse causation	Defined outcomes prior to the exposures

For peer review only

Reporting checklist for protocol of a systematic review.

Based on the PRISMA-P guidelines.

Instructions to authors

Complete this checklist by entering the page numbers from your manuscript where readers will find each of the items listed below.

Your article may not currently address all the items on the checklist. Please modify your text to include the missing information. If you are certain that an item does not apply, please write "n/a" and provide a short explanation.

Upload your completed checklist as an extra file when you submit to a journal.

In your methods section, say that you used the PRISMA-Preporting guidelines, and cite them as:

Moher D, Shamseer L, Clarke M, Ghersi D, Liberati A, Petticrew M, Shekelle P, Stewart LA. Preferred Reporting Items for Systematic Review and Meta-Analysis Protocols (PRISMA-P) 2015 statement. Syst Rev. 2015;4(1):1.

		Reporting Item	Page Number
**Title**			
Identification	#1a	Identify the report as a protocol of a systematic review	1
Update	#1b	If the protocol is for an update of a previous systematic review, identify as such	n/a

For peer review only

**Registration**			
	#2	If registered, provide the name of the registry (such as PROSPERO) and registration number	4
**Authors**			
Contact	#3a	Provide name, institutional affiliation, e-mail address of all protocol authors; provide physical mailing address of corresponding author	1
Contribution	#3b	Describe contributions of protocol authors and identify the guarantor of the review	15
**Amendments**			
	#4	If the protocol represents an amendment of a previously completed or published protocol, identify as such and list changes; otherwise, state plan for documenting important protocol amendments	n/a
**Support**			
Sources	#5a	Indicate sources of financial or other support for the review	18
Sponsor	#5b	Provide name for the review funder and / or sponsor	18
Role of sponsor or funder	#5c	Describe roles of funder(s), sponsor(s), and / or institution(s), if any, in developing the protocol	n/a
**Introduction**			
Rationale	#6	Describe the rationale for the review in the context of what is	6

For peer review only

		already known	
Objectives	#7	Provide an explicit statement of the question(s) the review will address with reference to participants, interventions, comparators, and outcomes (PICO)	7
**Methods**			
Eligibility criteria	#8	Specify the study characteristics (such as PICO, study design, setting, time frame) and report characteristics (such as years considered, language, publication status) to be used as criteria for eligibility for the review	8
Information sources	#9	Describe all intended information sources (such as electronic databases, contact with study authors, trial registers or other grey literature sources) with planned dates of coverage	10
Search strategy	#10	Present draft of search strategy to be used for at least one electronic database, including planned limits, such that it could be repeated	10
Study records - data management	#11a	Describe the mechanism(s) that will be used to manage records and data throughout the review	10
Study records - selection process	#11b	State the process that will be used for selecting studies (such as two independent reviewers) through each phase of the review (that is, screening, eligibility and inclusion in meta-analysis)	11
Study records - data collection	#11c	Describe planned method of extracting data from reports (such as piloting forms, done independently, in duplicate), any	11

For peer review only

process		processes for obtaining and confirming data from investigators	
Data items	#12	List and define all variables for which data will be sought (such as PICO items, funding sources), any pre-planned data assumptions and simplifications	11
Outcomes and prioritization	#13	List and define all outcomes for which data will be sought, including prioritization of main and additional outcomes, with rationale	12
Risk of bias in individual studies	#14	Describe anticipated methods for assessing risk of bias of individual studies, including whether this will be done at the outcome or study level, or both; state how this information will be used in data synthesis	12
Data synthesis	#15a	Describe criteria under which study data will be quantitatively synthesised	13
Data synthesis	#15b	If data are appropriate for quantitative synthesis, describe planned summary measures, methods of handling data and methods of combining data from studies, including any planned exploration of consistency (such as I2, Kendall's τ)	13
Data synthesis	#15c	Describe any proposed additional analyses (such as sensitivity or subgroup analyses, meta-regression)	13
Data synthesis	#15d	If quantitative synthesis is not appropriate, describe the type of summary planned	13
Meta-bias(es)	#16	Specify any planned assessment of meta-bias(es) (such as publication bias across studies, selective reporting within	13

For peer review only

		studies)	
Confidence in cumulative evidence	#17	Describe how the strength of the body of evidence will be assessed (such as GRADE)	13

The PRISMA-P checklist is distributed under the terms of the Creative Commons Attribution License CC-BY 4.0. This checklist was completed on 22. May 2019 using https://www.goodreports.org/, a tool made by the EQUATOR Network in collaboration with Penelope.ai

For peer review only
